# Simultaneous Determination of Multi-Class Pesticide Residues and PAHs in Plant Material and Soil Samples Using the Optimized QuEChERS Method and Tandem Mass Spectrometry Analysis

**DOI:** 10.3390/molecules27072140

**Published:** 2022-03-25

**Authors:** Magdalena Słowik-Borowiec, Ewa Szpyrka, Paulina Książek-Trela, Magdalena Podbielska

**Affiliations:** Department of Biotechnology, Institute of Biology and Biotechnology, Collegium of Natural Sciences, University of Rzeszow, 1 Pigoń St., 35-310 Rzeszow, Poland; ewaszpyrka@interia.pl (E.S.); pksiazek@ur.edu.pl (P.K.-T.); mpodbielska@ur.edu.pl (M.P.)

**Keywords:** pesticide residues, PAHs, mass spectrometry, validation, gas chromatography, QuEChERS

## Abstract

New analytical approaches to the simultaneous identification and quantification of 94 pesticides and 13 polycyclic aromatic hydrocarbons (PAHs) in five representative matrices (pepper, apple, lettuce, wheat, and soil) were developed. The analyses were based on gas chromatography coupled with triple quadrupole tandem mass spectrometry (GC-MS/MS). The procedure was optimized by changing the solvent used during the extraction, from acetonitrile to the acetone: *n*-hexane mixture at a volume ratio of 1:4 (*v*/*v*), as well as the use of a reduced amount of water during the extraction of compounds from cereals. An additional modification was the use of florisil instead of GCB in the sample cleanup step. A full method validation study was performed, at two concentration levels (LOQ and 1000 × LOQ), which showed satisfactory results for all analytes from the PAHs group, with recoveries ranging from 70.7–115.1%, and an average RSD of 3.9%. Linearity was tested in the range of 0.001–1.000 mg/kg and showed coefficients of determination (R^2^) ≥ 0.99 for all PAHs. Satisfactory recovery and precision parameters (LOQ and 100 × LOQ) were achieved for almost all analytes from the pesticide group in the range of 70.1–119.3% with the mean RSD equal to 5.9%. The observed linearity for all analytes in the concentration range of 0.005–1.44 mg/kg was R^2^ ≥ 0.99, with the exception of famoxadone, chizalofop-p-ethyl, prothioconazole, spirodiclofen, tefluthrin, and zoxamid. The extended uncertainties were estimated, using a top-down approach of 9.9% (average) and 15.3% (average) for PAHs and pesticides samples, respectively (the coverage factor k = 2, the 95% confidence level). Ultimately, the method was successfully applied to determine pesticide residues in commercial samples of fruit, vegetables and grain, and soil samples for PAHs, which were collected from selected places in the Podkarpacie region. A total of 38 real samples were tested, in which 10 pesticides and 13 PAHs were determined. Proposed changes allow us to shorten the sample preparation time (by 20%) and to reduce the consumption of organic solvents (by 17%). The use of florisil for sample cleanup, instead of GCB, improves the recovery of compounds with flat particles.

## 1. Introduction

Food quality and safety is becoming an increasingly important issue for consumers worldwide. Equally important topics concern environmental pollution, including soil, water, and air pollution with chemical agents. The presence of pesticides and polycyclic aromatic hydrocarbons (PAHs) in the environment is closely associated with human activity. The first of those compounds are related to the use of plant protection products in the agriculture, while the presence of the second group of substances results from human industrial activities.

Pesticides are compounds widely used to control pests and stabilize plant production [1]. Naturally, these effects provide many benefits, however, the unskillful or excessive use of these chemicals can be harmful not only to consumers’ health but also to the environment, and it is associated with the presence of pesticide residues. Pesticides cover an extensive range of chemicals, which are classified on the basis of their active ingredients, chemical structure, mode of action and toxicity. Their action is based on a disruption of: the synthesis of amino acids and proteins in weeds, the nervous system function in insects, cell division, energy production, respiration, growth regulation or development of photosynthesis, the deoxyribonucleic acid (DNA) methylation, and other effects [2,3]. Due to the high persistence of pesticides in the environment, their high biological activity, and a wide spectrum of toxic activity, these substances must be subject to strict legal regulations. Many countries and international organizations have defined Maximum Residue Levels (MRLs) of pesticides for foodstuffs, and in the European Union they are based on Commission Regulation (EC) No. 396/2005, as amended [4].

PAHs are common environmental pollutants that can come from various processes of incomplete combustion and pyrolysis. PAHs and their derivatives belong to a large class of organic compounds whose molecules consist of at least two condensed aromatic rings with a planar spatial structure. Those compounds that contain five or more aromatic rings are called heavy PAHs, while those containing less than five rings are light PAHs [5]. These substances are considered to be highly hazardous as they exhibit genotoxic and carcinogenic properties in the human body [6,7]. Numerous studies showed that these substances are found in food [8,9,10,11], and this may be caused by air and water pollution, the soil up taking, or thermal processing of food and raw products. In order to minimize the risks associated with the consumption of food containing PAHs, monitoring of contamination with these substances is essential; therefore, MRLs were established for these chemicals and included in the Commission Regulation (EC) No 1881/2006 [12], the Commission Regulation (EU) No 835/2011 [13] and the Regulation of the Minister of the Environment, 2016 [14].

In consequence, the analysis of trace amounts of pesticides and PAHs in food, foodstuffs and environmental samples has become essential. Currently, efforts are focused on developing very flexible analytical methods that would enable the determination of as many substances as possible in one analytical process, which are cheaper (allowing a reduction in the amount of solvents used) and, of course, environmentally friendly (eliminating toxic reagents and replacing them with less harmful ones). An equally important aspect in light of modern methods is also the shorter preparation time of the sample and further analysis using instrumental methods.

The procedure for the determination of pesticides and PAHs consists of many successive stages, including sampling, extraction, purification, and identification and quantification of tested substances. Proper sample preparation has a great influence on the quality and reliability of the obtained results [15].

In recent years, many studies on the selection of appropriate methods for the analysis of pesticides and PAHs, especially in food and soil, have been published (Table 1).

The most commonly used extraction and purification methods in the analysis of pesticide residues and PAHs are extraction with such reagents as acetonitrile, dichloromethane, ethyl acetate, methanol, as well as combinations, like acetonitrile-water or acetone-*n*-hexane (1:1), and a wide spectrum of salts and sorbents (Table 1), which effectively remove many co-extracted components from the complex sample matrix, such as dyes, fats, sugars, polyphenols, organic acids, and other. These contaminants could hinder the precise quantitative and qualitative course of the analysis [16,20]. The selection of the sample preparation process is closely related to the type of analyzed product and tested substances, including their physico-chemical properties. The QuEChERS concept has still remained a very popular method in recent years, and it is subject to numerous modifications and optimizations. The most popular instrumental methods for the determination of pesticide residues and PAHs are gas and liquid chromatography, most often coupled with mass spectrometry (Table 1).

In this work, the effectiveness and efficiency of the modified method based on the QuEChERS procedure [33], followed by gas chromatography coupled with triple quadrupole tandem mass spectrometry (GC-MS/MS) for the simultaneous determination of 94 pesticide residues and 13 substances from the PAH group in samples of plant products and soil were assessed. A detailed validation study was performed in order to assess fitness for purpose of the proposed method. Furthermore, to demonstrate the suitability of the optimized method, real samples were screened for pesticide residues and PAHs.

## 2. Results and Discussion

In this study, an alternative procedure of preparing samples for the analysis of pesticide residues and/or PAHs by gas chromatography and/or mass spectrometry in plant material and soil was elaborated. In order to assess the usefulness of the proposed method, validation was carried out with the determination of such parameters as: linearity, recovery, precision, limits of quantification, the working range of the method, and its uncertainty. The validation procedure was performed in accordance with the guidelines of the European Commission contained in the documents: SANTE [34] and the Commission Regulation (EU) [35].

In the present study, 94 pesticides (and their isomers) and 13 PAHs were selected for validation experiments. All included substances were amenable to the GC analysis, and were currently recommended in Polish agriculture or can be potentially used for protection of crops against pests and diseases. The study also covered persistent organochlorine pesticides and PAHs, commonly known as environmental contaminants. All selected analytes represent various structure classes, and they are characterized by highly varied physico-chemical properties.

In order to verify the suitability of the developed method for a specific purpose, it was used to determine the concentration of pesticide residues and PAHs in real samples.

## 3. Method Optimization and Validation

Samples of plant material, or other samples containing biological material, are associated with the diversity and complexity of matrices. Due to low concentrations of pesticide residues or PAHs in the samples, the critical stage is the appropriate preparation of sample extracts and reduction in interferences, to improve the analysis parameters [15]. The quantitative and qualitative assessment of the discussed compounds is very difficult and demanding, therefore the analytical methods used for this purpose are constantly developed. Today, specific and selective analytical methods play an important role in ensuring the correct and reliable determination of compounds, especially pesticide residues in food of plant origin, and they include the QuEChERS method combined with GC and HPLC chromatographic techniques with the MS and MS/MS mass spectrometry detector [36].

Validation studies were performed for four representative plant matrices: apple (from the group of plants with high water content, for which the preferred extract purification is conducted with primary and secondary amines (PSA)), wheat (from the group of cereal and seed matrices, purification with PSA and silica gel modified with octadecyl groups (C18EC)), peppers (from the group of pigment matrices, purification with primary and secondary amines (PSA) and florisil for PAHs or GCB for pesticides), and lettuce (from the group of highly pigmented matrices, purification with primary and secondary amines (PSA) and florisil for PAHs or GCB for pesticides), and for the soil (PSA treatment) (Table 2). Studies were carried out to assess the effectiveness of extraction and cleanup of the 94 target pesticides and 13 PAHs with different physical and chemical properties at two spiking levels (LOQ and 100 × LOQ for pesticides and LOQ and 1000 × LOQ for PAHs) (Details in Table 3 and Table 4). Plant and soil samples were prepared according to the optimized QuEChERS procedure. For the discussed samples, a full validation study in terms of evaluation of linearity, recovery, precision, as well as estimation of measurement uncertainty was performed. Details of the validation parameters are presented in Table 3 and Table 4.

Linearity was evaluated by studying five-level calibration curves plotted for pesticide and PAH standards prepared in blank extracts of the tested matrix, over a concentration range of 0.001–1.000 mg/kg (PAHs) and 0.005–1.44 mg/kg (pesticides). The linearity parameters were excellent with coefficients of determination (R^2^) ≥ 0.99 for all tested PAHs and almost all pesticides, except for famoxadone, chizalofop-p-ethyl, prothioconazole, spirodiclofen, tefluthrin and zoxamid. Detailed linearity data (linearity range, and R^2^ values) can be found in Table 3 and Table 4.

Obtained limits of detection (LOD) values ranged from 0.0002 to 0.005 mg/kg and 0.0003 mg/kg for pesticide and PAHs, respectively. Limits of quantification (LOQ) were set at the lowest spiking concentrations, and they were verified by recovery tests (n = 5) and the estimation of the relative standard deviation (RSD) for the obtained results in the tested matrix. Satisfactory recovery and precision parameters were obtained for all validated PAHs and for almost all pesticides (88 of 94 active substances) at this lowest level. These values were within the range of 0.001 mg/kg and 0.005–0.014 mg/kg for PAHs and pesticides, respectively. The highest spiking level for the tested samples was 1.000 mg/kg for PAHs and from 0.5 to 1.44 mg/kg for pesticide residues. All data have been shown in Table 3 and Table 4. Nine chromatograms for samples of a different matrix were added in Supplementary Material (Figures S1–S9).

Trueness and precision in terms of mean recovery and RSD were assessed for the proposed method by performing recovery experiments at two concentrations—these levels corresponded to the lowest and highest values of the linear range. The analysis of the spiked samples was performed in five replications at each level, using matched standards prepared in the apple, lettuce, wheat, pepper and soil extracts. The mean recovery of PAHs in samples ranged from 70.7–115.1% with an average RSD of 3.9% (for the soil matrix 71–100%, RSD 0.2–7.5%; cereals 82–109%; RSD 0.2–4.1%; apple 79–115%, RSD 0.4–9.0%; lettuce 93–115%, RSD 1.3–13.7%; and pepper 76–115%, RSD 1.3–7.3%) (Table 4). These values meet the requirements of European recommendations—the recovery was within the range of 50–120% [35]. On the other hand, for pesticide residues, the average recovery was from 70.1–119.3% with the mean RSD equal to 5.9%, with 70.6–116.7%, RSD 0.6–15.4% for soil matrix; 73.0–119.3%, RSD 0.4–18.2% for cereals; 70.1–118.5%, RSD 0.4–14.8% for apple; 72.2–109.5%, RSD 0.2–14.3% for lettuce; and 71.9–111.9%, RSD 0.3–11.3% for pepper. These values met the requirements of the European SANTE recommendations [34], with the recovery ranging from 70–120%, with a relative standard deviation (RSD) of ≤20% (Table 3).

The working range of the method was established by determining the limit of quantification and the highest level of spiking, whose acceptance criteria for accuracy, precision and linearity were met (Table 3 and Table 4).

The expanded measurement uncertainty was estimated by identifying all possible sources of uncertainty throughout the analytical process and calculating the uncertainty associated with each of them, following the ‘‘top-down’’ experimental approach [37]. The results showed that repeatability and recovery are the most important sources of uncertainty. The remaining components of the uncertainty budget, such as uncertainty related to weighing, dilution of standards, and purity of standards, are below 2% in the optimized method. The expanded uncertainty was calculated by multiplying the complex standard uncertainty by the coverage factor k. A probability of 95% for the coverage factor k = 2 was assumed. Uncertainty ranged from 4.2 to 18.8%, with an average uncertainty of 9.9% and from 4.2 to 31.4% with an average uncertainty of 15.3% for PAHs and pesticides, respectively (Table 3 and Table 4). According to the SANTE document [34] the uncertainty criterion for pesticide analysis is 50%.

The proposed modification of the PN–EN 15662: 2018 [33] standard method, known as the QuEChERS procedure, consists of changing the solvent used during the extraction from acetonitrile to the acetone: *n*-hexane mixture at a volume ratio of 1:4 (*v*/*v*). This change eliminates the last stage of sample preparation consisting of evaporation of acetonitrile immediately before the pesticides/PAHs analysis by gas chromatography and/or mass spectrometry, which in turn allows us to shorten the sample preparation time (by 20%) and to reduce the consumption of organic solvents (from 12 mL up to 10 mL per sample, i.e., by 17%). Moreover, it should also be noted that this method enables the simultaneous preparation of sample extracts both for the analysis of PAHs and pesticide residues (the standard method applies only to pesticides).

The use of a reduced amount of water during the extraction of compounds from cereals ensures a better separation of the organic and inorganic phases. On the other hand, in the case of soil samples, a hydration step was necessary (due to the very low water content in the samples). The addition of 10 mL of water did not reduce the extraction efficiency or affect the accuracy of the results. For both the soil and wheat matrices, cold water was added to the samples to compensate for the effect of the exothermic reaction (after adding magnesium sulfate at the extraction step) and loss of thermolabile compounds [38].

On the other hand, the use of florisil at the stage of sample cleanup (recommended for pigment and high-pigment matrices in the QuEChERS method), instead of the commonly used GCB, improves recovery (with an increase from 10% to 100% in the case of lettuce and peppers) of compounds with flat particles that are adsorbed on the surface of carbon (GCB). The effectiveness of replacing GCB with florisil, leading to better validation results, has already been confirmed in previous studies [39].

The use of an extraction mixture consisting of acetone and *n*-hexane (1:4 *v*/*v*) enables elimination of a more toxic organic solvent, i.e., acetonitrile (NDS of 600 mg/m^3^, 70 mg/m^3^, and 72 mg/m^3^ for acetone, acetonitrile, and hexane, respectively).

The authors of the works related to the development of methods for the determination of PAHs and pesticide residues, both separately and simultaneously, were guided by the basic issues, such as: selection and amount of the sample, type of extraction solvent, pH effect, type and amount of salt and sorbents used in the purification phase and the type of analyte separation technique (Table 1). The advantage of the most popular extraction solvent, acetonitrile, is its compatibility to chromatographic applications, although it interferes with specific GC detectors, e.g., for nitrogen, and is definitely less volatile than other organic solvents, which may extend the evaporation and concentration stage [40,41]. Moreover, due to the relatively low solubility of lipids in acetonitrile, the co-extraction of lipids with this solvent is quite low, however, there may be limitations concerning the availability of pesticides from lipids and loss of non-polar pesticides [40]. Other non-halogenated solvents used, such as acetone, ethyl acetate or hexane, are much less polar in relation to acetonitrile; therefore, polar pesticides of medium and high polarity have a much better solubility in acetonitrile and thus higher recoveries when it is used for extraction. In addition, ethyl acetate is also characterized by the ability to extract lipids and waxes, and gives lower recoveries when compared to acid/alkaline pesticides and generally lower purification efficiency in DSPE [42]. Extraction with hexane is more characteristic for hydrophobic components in aqueous matrices, and for very low extraction of polar matrix components (proteins, amino acids, carbohydrates, etc.) [43].

Table 1 shows examples of sorbents and salts used in the purification stage. The most frequently used compounds were PSA (primary secondary amine), octadecyl sorbent C18, SAX (quaternary amine), Z-Sep (sorbent based on modified silica gel with oxide zirconium) and GCB (graphitized carbon black) [16,17,20,25,30]. These are sorbents that are responsible for the removal of polar organic acids, some sugars, lipids and sterols, carotenoids and chlorophyll [44]. Other commercially available sorbents are Z-Sep Plus (is a sorbent based on modified silica gel with zirconium oxide dual bonded on the same silica particles) [45,46], EMR-Lipid (“enhanced matrix removal” to selectively remove lipids from extracts of fatty foods) [47,48], ChloroFiltr (polymetric sorbent to removal of chlorophyll) [49] and ENVI-Carb (is used to eliminate polar compounds, pigments and polyphenols) [50]. In case of flat molecules, the use of GCB introduces strong limitations in the recovery of analytes, which was also confirmed in our research. For this reason we decided to replace this sorbent with florisil. This change resulted in a significant improvement in recoveries (from 10% to 100%). Generally, florisil is magnesium silicate SiO_2_ + MgO, a polar compound classified as amphoteric. It is intended to be used for the isolation of hydrophilic polar substances from non-aqueous, non-polar mixtures, for analyses of samples with a high content of lipids, waxes or oils, for adsorption of pesticides from environmental samples, or for separation of aromatic compounds from aliphatic-aromatic mixtures, as well as for similar applications. [51]. The salts used for the extraction and purification, or their combinations (MgSO_4_, NaCl, Na_2_SO_4_) enable a good separation of the aqueous and organic phases, as well as better recoveries, especially of polar compounds, which may otherwise be lost in the aqueous phase [39,52]. On the other hand, the compounds used in the described procedures, trisodium citrate dihydrate and disodium hydrogen citrate sesquihydrate, affect the recovery of acid-labile analytes [44].

The last steps in the procedure for the determination of PAHs and pesticides involves instrumental analysis, most often conducted using the HPLC-MS/MS, GC-MS/M, or LC-MS/MS systems (Table 1), and which enable the analysis of the components within the desired detection limits. The limits of quantification determined in this validation experiment for the tested matrices are much lower than the maximum permissible levels (MRLs) specified in the Regulations for individual analytes, therefore the method can be used for routine tests.

In conclusion, the possibility of introducing new methods or modifying the existing ones, on a basis of the use of various solvents, salts, buffers and sorbents, enables the implementation of a wide spectrum of analyzed analytes and matrices in the analysis of pesticides and PAHs.

## 4. Real Samples

In order to demonstrate the suitability of the optimized method for routine quantifications, real samples were screened for pesticide and PAHs residues. The study included 28 samples of plant material: apple (n = 8), wheat (n = 7), lettuce (n = 6), and pepper (n = 7) (Table 5), which were purchased at the local commercial shops, as well as 10 samples of soil. Soil samples came from the Podkarpacie region (Poland) and were collected from a city center, a housing estate, recreation areas, banks of a river (San), farmland, railway tracks, sewage treatment plants, power plants, and steelworks, as detailed in Table 6.

The results of the analyses concerning pesticide residues were interpreted by comparing the MRL values in force in Poland [4] and by verifying the correct use of chemical preparations on the basis of the current “Register of plant protection products authorized for marketing and use” [14] and “Labels-instructions for the use of plant protection products” approved for marketing and use with the permit of the Ministry of Agriculture [53]. 17.5% (five samples) were free of pesticide residues, while the majority of samples, in which residues were found, contained more than one pesticide (two active substances detected in seven samples (25%), and three active substances found in two samples (7%). Most frequently, the determined pesticide residues belonged to fungicides, with boscalid being the most frequently detected compound. Four apple samples exceeded the established MRLs for boscalid. However, there was no violation of the law concerning the use of plant protection products not recommended for a given crop in any of the samples. The test results are shown in Table 5, where the MRL values for individual substances are also given.

Table 6, which concerns the analysis of soil samples for PAHs, shows that the least contaminated material were samples two, three and seven, which came from a housing estate, an arable field and the San River bank, respectively. In samples two and three, all determined compounds were < LOQ, and in sample seven, only two compounds above the LOQ were detected, i.e., phenanthrene and pyrene, at a level of 0.001 mg/kg. On the other hand, the most polluted samples were from the steelworks (No. five), the sewage treatment plant (No. six) and the city center (No. eight). The highest level of pyrene pollution, 0.092 mg/kg, was recorded in the smelter, and of indeo (one, two, three) pyrene in the city center, also at a level of 0.092 mg/kg. Furthermore, the results presented in the table show that the most frequently detected compounds from the PAH group were phenanthrene and pyrene, and acenaphthylene was not detected above the LOQ in any collected material. Examples of chromatograms of real samples are presented in the Supplementary Material (Figures S3, S6 and S9).

The obtained results of actual soil samples were compared to the permissible PAH content in the soil according to the Minister of the Environment [54,55], depending on a relevant soil type applicable to a given soil. The data are presented in Table 6.

From a review of scientific publications it can be concluded that the presence of PAHs or pesticides is common, and in many cases the concentration levels of these substances exceed the permissible MRLs.

Kubecki, M., (2010) [56], Majkowska, E. et al., (2016) [57], and Wang, D., et al. (2018) [28] showed a relationship between the location of the sampling site and the occurrence of cases of exceeded levels for these substances. It is worth remembering that the problem of hydrocarbon pollution also affects the food we eat. The surprising results on PAHs concentrations in food were published by EFSA under the Commission Order 2005/108/EC. The obtained data showed that 47.3% of 1000 tested samples were above the detection limit for the presence of benzo(a)pyrene [58,59].

In case of pesticide residues, there are also reports on the monitoring of these substances. In our previous work [60] we showed the presence of pesticides in agricultural products from south-eastern Poland. The residues of these xenobiotics were detected in 84 (25.6%) of the analyzed samples, and in seven samples (2.1%) they exceeded the MRL level. Most often, pesticide residues were detected in samples of fruit, herbs, and vegetables.

The problem of pesticide residues in food and soil is common, and this is also confirmed by other publications: 35 active substances were found in vegetables and fruit (lettuces, oranges, peppers, tomatoes, and carrots) [23], 93 pesticides in flour wheat, lettuce and apples, 13 pesticides in soil and water [61], 105 pesticides in cereals [62], and 35 pesticides of various classes in tropical fruit [30]. Additionally, the European Food Safety Authority publishes numerous scientific papers or reports [63,64] on the presence of pesticide residues in food, which further highlights this problem. On a basis of the above information, it can definitely be concluded that it is necessary to continuously monitor pesticide residues and PAHs, as well as to implement appropriate measures to prevent overexposure to these substances.

## 5. Materials and Methods

### 5.1. Chemicals and Standards

Acetone and *n*-hexane of high purity were purchased from Honeywell Specialty Chemicals Seelze GmbH (Germany). Kits of salt and sorbents (SampliQ) for extraction and purification (Table 4) were obtained from Agilent Technologies (Santa Clara, CA, USA). Florisil was purchased from Sigma-Aldrich Chemie GmbH (Saint Louis, MO, USA). Water for sample preparations was purified through SolPure XIO P (ELKAR, Kęty, Poland).

The individual pesticide analytical standard (94 active substances) were obtained from Dr. Ehrenstorfer (Augsburg, Germany), Supelco^®^ (Bellefonte, PA, USA) and the Institute of Organic Industry (IPO) (Warsaw, Poland). A certified mixture of 13 PAHs standard solutions in acetone (EPA 525 PAHs Mix B) was purchased from Supelco^®^ (Bellefonte, PA, USA). Triphenyl phosphate (TPP) was used as the internal standard (IS), and it was obtained from Dr. Ehrenstorfer (Augsburg, Germany).

All analytical standard compounds were of >98% purity, and they were prepared in concentrations of about 1000 µg/mL in acetone, then the intermediate stock standard mixture at approximately 10 µg/mL (of each compound) was prepared from the stock solutions by dilution in acetone. Subsequent working standards mixtures of 0.001–1.00 µg/mL (for PAHs) and 0.005–1.44 µg/mL (for pesticide) were prepared in an acetone: *n*-hexane (1:4 *v*/*v*). The IS was prepared by dissolving the TPP in acetone: *n*-hexane (1:4 *v*/*v*) to obtain a 1000 µg/mL solution. Calibration of matrix-matched standards was performed by mixing working standards solutions with blank sample extracts containing one g of sample per one mL of solvent. The pesticide and PAHs standards at appropriate concentrations were used to calibrate the GC-MS/MS system and spike samples in validation experiments. The mixture of analytical standards, working solutions and IS were stored in glass bottles closed with parafilm at 4 °C in dark pending the analysis.

### 5.2. Sample Preparation Procedure

The method for preparing samples for the analysis of pesticide residues and/or PAHs by gas chromatography with mass spectrometry in plant material and soil was a modification of the standardized method provided in PN-EN 15662: 2018-06. A Vegetable Food Multimeter was used to determine pesticide residues using GC and LC based analysis after acetonitrile extraction/partitioning and dispersive solid phase extraction (DSPE), as described in QuEChERS modular method [33]. The proposed solution is to change the solvent used during the extraction from acetonitrile to the acetone: *n*-hexane mixture at a volume ratio of 1:4 (*v*/*v*). An additional change in the procedure was the use of a reduced amount of water during the extraction of compounds from cereals, and the use of florisil instead of GCB at the sample cleanup step, as it prevents the loss of planar compounds such as PAH. The quoted methodological standard does not describe the procedure for the extraction and cleanup of soil samples for the determination of PAHs and pesticides. In this work, as a novelty, a solution dedicated to this type of matrix was proposed.

### 5.3. The Sample Preparation Scheme Is as Follows

Samples of plant material or soil (of five g or ten g depending on the matrix; Table 2) with water added, if necessary (Table 2), were extracted with 10 mL mixture of acetone: *n*-hexane (1:4 *v*/*v*) for approx. 1 min (vortex shaking; Vortex BenchMixerTM, Benchmark, Edison, NY, USA). Next, buffer salts were added containing: four g of anhydrous magnesium sulfate (MgSO_4_), one g of sodium chloride (NaCl), one g of anhydrous trisodium citrate, and 0.5 g of disodium sesquihydrate citrate, and then samples were shaken again vigorously for one min. In the next step, the samples were centrifuged at the centrifuge speed > 4000 rpm (5804R, Eppendorf, Hamburg, Germany) for five min. The next step was the cleanup of the samples using the dispersive solid phase extraction (DSPE). For this purpose five mL of sample extract (upper organic layer) were taken and extracted with the mixture of salts I, II, III or IV (depending on the matrix; Table 2). The samples were shaken vigorously for approx. 30 s (for mixtures of salts I and II) or approx. two min (for mixtures of salts III and IV), and then the samples were centrifuged at the centrifuge speed > 4000 rpm for five min.

The analysis of each sample was performed in five replications, with IS at a concentration of 1000 µg/mL in the amount of 50 µL added to each of them immediately before the chromatographic analysis. The sample extracts obtained this way were ready for analysis by GC-ECD/NPD, GC-MS or GC-MS/MS.

### 5.4. Chromatographic Analysis

A 7890A gas chromatograph (Agilent Technologies, USA) gas chromatography coupled with triple quadrupole tandem mass spectrometry, model 7000 (GC-MS/MS), was used to analyze sample extracts. Chromatographic separations were conducted using the HP-5 MS Ultra Inert column (30 m × 0.25 mm I.D.× 0.25-μm; Agilent Technologies, USA). Analyses were conducted in the selected ion monitoring mode (SIM) based on the use of one of the quantitative ions for determination of PAHs and the multiple reaction monitoring (MRM) mode with three mass transitions for each pesticide. Analyzed compounds were identified according to their qualitative ions and retention times. The following analysis parameters were used: samples injected in a splitless mode, injected volume—2 μL, carrier gas—helium (5.0 purity, flow 2.1 mL/min), the MS ionization was carried out in the electron ionization mode at 70 eV. For pesticide residues, the temperature was 280 °C for the transfer line, 300 °C for the ion source, 150 °C for the quadrupoles, and 70–280 °C for the oven. For PAHs analysis, the temperature was 320 °C for the transfer line, 320 °C for the ion source, 150 °C for the quadrupoles, and 80–320 °C for the oven. Software Mass Hunter, version B.07.06, was used for data acquisition, control and data processing of the analysis results. MRM transition, SIM and retention times of tested substances can be found in the Supplementary Material (Tables S1 and S2) attached to this article. The total run time was 42 and 26 min for pesticide and PAHs, respectively.

### 5.5. Validation Process

In order to determine the usefulness of the developed method, a validation was carried out with the parameters of linearity, recovery, precision, and limits of detection and quantification assessed, and measurement of uncertainty. The validation procedure was performed in accordance with the European Commission guidelines specified in Guidance SANTE/12682/2019—Guidance document on analytical quality control and method validation procedures for pesticides residues analysis in food and feed [34] and Commission Regulation (EU) (2011) No 836/2011 of 19 August 2011 amending Regulation (EC) No 333/2007 laying down the methods of sampling and analysis for the official control of the levels of lead, cadmium, mercury, inorganic tin, 3-MCPD and benzo(a)pyrene in food stuffs [35].

Validation studies were performed for four representative plant matrices: apple, wheat, pepper, lettuce, and for soil (Table 2).

Linearity was studied by GC-MS/MS analysis at five different concentration levels over the ranges of 0.005–1.440 μg/mL and 0.001–1.000 μg/mL for pesticide residues and PAHs, respectively. Calibration standards were prepared in sample extracts, and they were applied in three repetitions per level over three days.

The limit of quantification (LOQ) was set at the lowest spiking concentration that has been validated with satisfactory recovery and precision parameters. Limits of detection (LOD) were calculated using a signal-to-noise ratio criteria, equal to three for all tested substances [34].

In the recovery experiments, samples were spiked with the appropriate volumes of working standard solutions of pesticides and PAHs, at two levels: LOQ and 1000 × LOQ. The samples were prepared using the method described above, and next they were analyzed for each spiking level in five replicates (n = 5). Precision was calculated from the recovery experiments, and it was expressed in terms of relative standard deviation (RSD%) at each spiking level. Recovery per level and overall recovery were determined for every tested substance. For quantitative methods, the established mean recovery for spiked samples should be between 70–120%, with a relative standard deviation (RSD) below or equal to 20% for pesticide residues and 50–120% for PAHs [35].

Uncertainty of measurement (U) was estimated on the basis of the results obtained in the validation process. The major uncertainty sources were the repeatability of recoveries from spiked samples and uncertainty of the average recovery calculated from the rectangular distribution. The relative expanded uncertainty was calculated by using the coverage factor k = 2 at the confidence level of 95% [65].

## 6. Conclusions

In this article, the sample preparation procedure (based on the QuEChERS method [33] has been optimized to separate pesticide residues and PAHs from five representative matrices and analysis by GC-MS/MS system. The novelty in this research was to change the solvent used during the extraction from acetonitrile to the acetone: n-hexane mixture at a volume ratio of 1:4 (*v*/*v*). An additional change was a reduction in the amount of water used during the extraction of compounds from cereals, and the use of florisil instead of GCB at the sample cleanup step. The method was validated for a total of 94 substances from the pesticide and 13 compounds from the PAH group. Overall linearity, precision, and accuracy parameters were highly satisfactory, with the exception of six pesticides. The extended uncertainty of the method was also acceptable. The proposed analytical procedure is efficient, accurate and repeatable, and therefore, is suitable for simultaneous determination of multiclass pesticide residues and PAHs in plant material and soil samples. It is also safer, allowing for a reduction in the consumption of organic solvents (by 17%) and time of sample preparation (by 20%). The use of florisil for sample cleanup, improves recovery of compounds with flat particles. The developed method was successfully used to test a total of 38 real soil and plant material samples. The results of the analysis of samples revealed cases of the MRLs violation for pesticides (the case of boscalid), however, no substances not approved for use in the tested crops were found. As for PAHs, no MRL exceedance was detected. Nevertheless, the obtained results indicate the need for the further monitoring of these compounds.

## Figures and Tables

**Table 1:** Examples of methods application in pesticides and PAHs analysis from various sample type.

Product Type	Analyzed Substance	Sample Preparation/ Extraction/Cleanup	Analysis	Analytical Scope	LOQ	RSD [%]	Recovery	References
Black, green, red and white tea	13 PAHs	QuEChERS/10 mL of water, 10 mL of ACN, 1 g of NaCl, 4 g of MgSO_4_/0.15 g of PSA, 0.15 g of SAX, 0.9 g of MgSO_4_,	GC–MS	0.1–100 ng/mL	<0.9 µg/kg	<20%	50–120%	[16]
Catfish	pesticides, PAHs, PCBs, PBDEs	4 mL of ACN/2 g of MgSO_4,_ 2 g of NaCl/45 mg of MgSO_4,_ PSA, C18, Z-Sep, Carbon X (20/8/8/8/1)	UHPLC–MS/MS, GC–MS/MS	5–40 ng/g	<5 ng/g	<20%	70–120%	[17]
Cucumber, grapefruit	233 pesticides	QuEChERS/15 mL of methanol-acetic acid (99:1, *v*/*v*), 6 g of MgSO_4_, 1.5 g of sodium acetate/900 mg of MgSO_4_, 150 of mg PSA	GC–MS, LC–MS/MS	5–160 µg/kg	0.13–11.80 µg/kg	<20%	77.87–104.15%	[18]
Daily food	18 PAHs	QuEChERS/5 mL of water, 10 mL of ACN, 4 g of MgSO_4,_ 1 g of NaCl/0.9 g of MgSO_4,_ 0.3 g of PSA, 0.3 g of C18	GC–MS/MS	1–10 µg/kg	0.03–0.6 µg/kg	<23%	70–101%	[19]
Fresh herbs: basil, tarragon, sage, lovage, mint, parsley, rosemary, oregano	27 pesticides, 7 PAHs	5 mL of water, 10 mL of ACN/1 g of NaCl, 4 g of MgSO_4,_ 1 g of trisodium citrate dihydrate, 0.5 g of disodium hydrogencitrate sesquihydrate/0.15 g of PSA, 0.05 g od GCB, 0.9 g of MgSO_4_	GC–MS	0–400 ng/mL	<12 µg/kg	<15%	71.6–116.9%	[20]
Honey	90 pesticides, 16 PAHs, 22 PCBs	SPME/10 mL of water, 10 mL of ACN, 1 g of NaCl, 4 g of MgSO_4,_ 1 g of trisodium citrate dihydrate, and 0.5 g of disodium hydrogencitrate sesquihydrate/1.2 g of MgSO_4_, 400 mg of primary–secondary amine (PSA), 400 mg of C18	GC–MS/MS, LC–MS/MS	10–3000 ng/g	60 ng/g	<20%	60–103%	[21]
Honey	161 pesticides, PCB, PBDE	10 mL of ACN:water (1:1), cooled for 20 min, 4 g of MgSO_4,_ 1 g of NaCl, 1 g of trisodium citrate dihydrate, and 0.5 g of disodium hydrogencitrate sesquihydrate/cooled overnight/60 mg of PSA, 50 mg of C18/filtered through nylon syringe filter, 30 µL of formic acid: ACN (5:95)	LC–MS, GC–MS	–	0.2–14 µg/kg	<20%	70–120%	[22]
Lettuces, oranges, peppers, tomatoes, carrots	35 pesticides	QuEChERS/15 mL of 1% (*v*/*v*) acetic acid in ACN, 6 g of MgSO_4_, 1.5 g of sodium acetate/150 mg of MgSO4, 50 mg of PSA, 50 mg of GCB	GC–MS	10–100 µg/kg	<10 µg/kg	–	78–113%	[23]
Napa cabbages, common beans, cucumbers, tomatoes, Chinese leeks, celery	16 PAHs	QuEChERS/30 mL of ACN, 4 g of MgSO_4_, 1 g of NaCl/purified by the dispersive solid-phase extraction (dSPE) method and concentrated in a water bath at 40°C until almost dry	GC–MS	0.1–10 µg/kg	0.04–0.1 ng/g	1–9%	71–108.2%	[24]
Peach, plum, pear, baby apple, strawberry, passion fruits while fresh vegetables include potato, cabbage, cauliflower, carrot, garlic, broccoli, leek, celery, ginger, peas, lettuce	5 organophosphate pesticides	10 mL of ACN/5 g of MgSO_4,_ 1.2 g of NaCl/9 mg of PSA, 9 mg of GCB, 100 mg of C18, 125 mg of MgSO_4_	LC–MS/MS	5–500 µg/L	0.5–5 µg/kg	13.26%	76.89–110.3%	[25]
Pineapple	86 multiclass pesticides	10 mL of ethyl acetate, 1.5 g of NaCl, 5 g of MgSO_4_/50 mg of PSA, 150 mg of Na_2_SO_4_	GC–MS/MS	10–100 ng/g	10 ng/g	<20%	70–120%	[26]
Smoked fish, smoked cheeses	16 PAHs	QuEChERS/10 mL of water, 10 mL of ACN, 6 g of MgSO_4_, 1.5 g of sodium acetate/400 mg of PSA, 400 mg of C18-silica, 1200 mg of MgSO_4_	GC–MS	0.1–1 ng/g	0.020–0.512 ng/g	–	35.8–103.4%	[27]
Soil	16 PAHs	20 mL of dichloromethane, 3 g of Na_2_SO_4_/150 mg of PSA, 50 mg of C18, 900 mg of Na_2_SO_4_	GC–MS	2–1000 µg/kg	–	–	65–119%	[28]
Tea, dry products	15 PAHs	SPME: 1 mL of methanol, sonication at 42 kHz	HPLC–FLD	0.05–2 ng/mL	0.21–3.08 ng/g	–	70–120%	[29]
Tropical fruit—rose apple/pomarrosa, starfruit/carambola, yoyomo, papayuela	35 multiclass pesticides	QuEChERS/15 mL of ACN containing 0.05% formic acid, 6 g of MgSO_4,_ 1.5 g of sodium acetate/150 mg of MgSO_4_, 50 mg of PSA, 50 mg of C18, 7.5 mg of GCB	GC–MS/MS	5–600 µg/kg	5 µg/kg	<20%	70–120%	[30]
Water, pear, tomato, cucumber, eggplant, cilantro	88 pesticides	QuEChERS/10 mL of ACN containing 4.4% formic acid, 5g of ammonium formate_,_ 1.5g of MgSO_4_/1.5g of MgSO_4_, 500mg of PSA, 500 mg of C18/500mg of PSA, 500 mg of C18	LC–MS/MS	10–100 ng/g	10 ng/g	<25%	70–120%	[31]
Wheat	28 PAHs, 15 pesticides	50 mL of acetone: *n-*hexane (1:1 *v*/*v*), filtered through filter paper, evaporated to dryness at 40° C, dissolved in 5 mL of cyclohexane-dichloromethane (1:1 *v*/*v*)	GC–MS/MS	0.1–5 µg/kg	0.02–0.07 µg/kg	3–15%	76–110%	[32]

polychlorinated biphenyls (PCBs), polybrominated diphenyl ethers (PBDEs), polycyclic aromatic hydrocarbons (PAHs), acetonitrile (ACN).

**Table 2:** List of salts and sorbents used for extraction and cleanup of individual matrices.

Products (Matrices)	Mixture for d-SPE	Sample Weight [g]	Water [mL]
Apple (fruit and vegetables)	I (150 mg of PSA, 900 mg of MgSO_4_)	10	–
Soil		5	10
Cereals (seeds, grains, fruit and vegetables with fat and wax content)	II (150 mg of PSA 150 mg of C18EC 900 mg of MgSO_4_)	5	5 (modification: reduction from 10 mL to 5 mL)
Peppers (pigmented fruit and vegetables (containing, among others, carotenoids and chlorophyll)	III a (150 mg of PSA 15 mg of GCB 900 mg of MgSO_4_) III b (150 mg of PSA 500 mg of florisil, 900 mg of MgSO_4_)	10	–
Lettuce (highly pigmented fruit and vegetables (high in carotenoids and chlorophyll)	IV a (150 mg of PSA 45 mg of GCB 900 (855) mg of MgSO_4_) IV b (150 mg of PSA 750 mg of florisil, 900 (855) mg of MgSO_4_)	10	–

a—salts used for the purification of sample extracts (pesticides); b—salts used for the purification of sample extracts (PAHs)—method modification—GCB replaced with florisil, which prevents the loss of planar compounds such as PAH.

**Table 3:** Validation parameters for pesticide residue determinations (linearity range, calibration curve and coefficient of determination, recovery, standard deviation from recoveries (RSD), measurement uncertainty (U)).

Compound	Matrix Type	Linearity Range [mg/kg]	LOD /LOQ (mg/kg)	R^2^	Recovery % (RSD, n = 5)		U (k = 2) [%]
					LOQ	100 × LOQ	
Acetamiprid	apple	0.010–0.99	0.003 /0.010	0.979	85.1 (9.9)	91.1 (3.4)	17.1
	soil			0.990	86.3 (12.5)	83.8 (0.8)	20.0
	wheat			0.997	108.4 (8.3)	101.1 (4.1)	12.4
	lettuce			0.997	79.6 (5.5)	81.1 (9.6)	22.2
	pepper			0.992	94.0 (8.5)	89.9 (7.9)	18.6
Acrinathrin	apple	0.010–1.00	0.003 /0.010	0.990	93.1 (6.0)	104.5 (4.9)	11.9
	soil			0.994	88.6 (6.5)	82.7 (5.5)	16.5
	wheat			0.997	104.3 (3.6)	105.5 (4.1)	8.2
	lettuce			0.994	84.5 (10.5)	109.5 (4.6)	18.4
	pepper			0.992	86.9 (6.2)	89.2 (8.1)	17.8
Aldrin	apple	0.013–1.270	0.004 /0.013	0.996	76.0 (4.3)	102.4 (5.0)	14.1
	soil			0.998	94.7 (9.6)	96.8 (2.2)	13.0
	wheat			1.000	94.2 (6.8)	104.0 (3.8)	11.5
	lettuce			0.999	91.8 (4.0)	93.0 (4.9)	10.9
	pepper			0.997	80.8 (4.8)	103.5 (3.8)	12.2
Azoxystrobin	apple	0.009–0.93	0.003 /0.009	0.988	80.2 (10.2)	105.2 (5.6)	19.6
	soil			0.992	93.1 (14.7)	84.9 (9.7)	28.2
	wheat			0.993	115.1 (7.5)	82.4 (13.8)	25.5
	lettuce			0.997	91.4 (6.0)	91.8 (5.9)	14.0
	pepper			0.998	85.9 (7.7)	74.9 (3.1)	18.3
Benalaxyl	apple	0.010–1.00	0.003 /0.010	1.000	76.8 (4.3)	94.3 (5.4)	14.8
	soil			0.997	89.1 (2.6)	70.7 (0.9)	13.1
	wheat			0.992	111.7 (6.5)	102.3 (1.6)	8.9
	lettuce			0.999	92.0 (5.5)	99.4 (6.5)	13.1
	pepper			0.991	89.4 (7.4)	98.3 (2.7)	11.9
Bifenazat	apple	0.010–1.00	0.003 /0.010	0.999	79.0 (3.3)	77.9 (9.5)	21.3
	soil			1.000	106.4 (3.8)	98.7 (7.0)	11.3
	wheat			0.994	74.5 (4.0)	96.4 (10.1)	19.7
	lettuce			0.999	92.7 (7.6)	80.4 (2.0)	14.8
	pepper			0.999	91.5 (6.4)	92.7 (2.9)	11.4
Boscalide	apple	0.010–1.00	0.003 /0.010	0.997	86.8 (12.9)	91.8 (7.0)	23.4
	soil			0.994	86.4 (1.5)	109.7 (4.3)	9.4
	wheat			1.000	94.3 (12.7)	108.7 (6.3)	20.0
	lettuce			0.993	88.8 (7.1)	78.0 (2.5)	15.9
	pepper			0.998	86.3 (8.3)	88.6 (2.3)	14.8
Bromuconazole	apple	0.010–1.00	0.003 /0.010	0.986	110.1 (9.4)	77.2 (9.3)	22.9
	soil			0.995	102.2 (8.8)	111.8 (4.1)	13.8
	wheat			0.994	79.9 (10.2)	117.2 (3.7)	20.1
	lettuce			1.000	101.5 (6.6)	82.7 (2.6)	12.7
	pepper			0.998	92.3 (6.9)	93.8 (6.1)	14.7
Bupirimate	apple	0.010–1.00	0.003 /0.010	0.999	81.7 (12.7)	98.5 (5.7)	22.4
	soil			1.000	106.4 (10.4)	91.9 (4.6)	15.7
	wheat			0.991	97.3 (12.4)	93.7 (10.6)	24.3
	lettuce			0.999	78.1 (6.0)	88.0 (1.8)	14.2
	pepper			1.000	79.6 (8.7)	98.7 (4.3)	17.0
Buprofezin	apple	0.010–1.00	0.003 /0.010	0.999	75.6 (5.3)	87.1 (5.9)	17.9
	soil			1.000	79.5 (12.5)	112.3 (3.8)	21.9
	wheat			0.998	106.9 (10.9)	113.0 (7.0)	17.8
	lettuce			1.000	81.5 (4.3)	92.8 (3.7)	12.3
	pepper			1.000	89.5 (4.4)	91.5 (2.2)	9.5
Captan	apple	0.010–0.98	0.003 /0.010	0.842	70.7 (0.7)	79.7 (7.4)	19.6
	soil			0.990	94.7 (2.4)	85.7 (10.9)	16.6
	wheat			0.978	100.5 (8.4)	98.2 (16.6)	25.4
	lettuce			0.999	83.3 (3.8)	97.3 (2.4)	9.6
	pepper			0.994	93.4 (5.9)	87.7 (6.8)	15.3
Clomazone	apple	0.014–1.44	0.005 /0.014	0.997	87.2 (6.0)	81.4 (9.4)	20.6
	soil			0.994	83.4 (8.7)	111.7 (7.7)	19.3
	wheat			0.994	85.3 (13.1)	117.5 (9.3)	25.4
	lettuce			1.000	96.1 (4.9)	87.7 (2.7)	10.2
	pepper			1.000	89.6 (3.6)	92.4 (3.4)	9.6
Chlorantraniliprole	apple	0.010–1.00	0.003 /0.010	0.988	82.8 (3.2)	103.7 (0.7)	8.2
	soil			0.996	87.1 (7.9)	86.0 (7.2)	19.2
	wheat			0.993	95.7 (2.3)	73.0 (2.8)	11.7
	lettuce			0.999	88.2 (6.9)	76.4 (2.9)	16.4
	pepper			0.994	87.2 (6.2)	93.2 (4.6)	13.5
Chlorprifos	apple	0.010–1.00	0.003 /0.010	0.994	89.5 (3.8)	105.6 (5.0)	10.5
	soil			0.997	95.0 (1.7)	106.2 (4.5)	7.3
	wheat			0.995	92.8 (8.3)	101.1 (8.7)	17.9
	lettuce			0.999	92.3 (3.2)	107.0 (5.6)	10.0
	pepper			0.999	91.3 (1.5)	94.0 (6.4)	10.4
Chlorpyrifos methyl	apple	0.010–1.00	0.003 /0.010	0.994	78.6 (2.1)	108.4 (3.1)	10.8
	soil			0.994	80.9 (4.8)	83.8 (12.4)	23.8
	wheat			0.998	99.0 (8.9)	86.2 (5.1)	16.2
	lettuce			1.000	96.3 (3.7)	86.9 (5.4)	11.5
	pepper			1.000	87.9 (7.6)	98.2 (4.5)	14.5
Cyflufenamid	apple	0.010–1.00	0.003 /0.010	0.991	107.6 (8.8)	92.4 (4.9)	14.4
	soil			0.997	78.5 (3.3)	115.5 (2.4)	12.7
	wheat			0.996	91.1 (14.1)	113.9 (1.4)	20.1
	lettuce			0.998	94.0 (8.9)	97.6 (4.9)	14.9
	pepper			0.998	77.0 (5.2)	93.9 (4.2)	14.5
Cypermethrin	apple	0.009–0.89	0.003 /0.009	0.993	79.0 (4.7)	92.5(7.6)	17.2
	soil			0.995	84.4 (13.0)	82.2 (1.2)	21.6
	wheat			0.993	94.9 (3.5)	91.7 (11.4)	16.8
	lettuce			0.994	88.6 (6.9)	89.8 (3.3)	13.4
	pepper			0.990	86.7 (7.4)	78.0 (5.7)	19.2
Cyprodinil	apple	0.010–1.00	0.003 /0.010	1.000	110.6 (4.7)	112.2 (3.6)	10.3
	soil			0.997	88.3 (6.4)	113.6 (6.6)	15.2
	wheat			0.999	104.7 (3.3)	110.4 (3.8)	8.3
	lettuce			1.000	94.2 (4.1)	89.9 (2.7)	9.1
	pepper			1.000	83.6 (5.4)	91.4 (3.5)	12.8
Cyproconazole	apple	0.010–1.01	0.003 /0.010	0.969	75.6 (7.6)	102.8 (2.9)	15.5
	soil			0.999	90.0 (5.5)	110.4 (8.3)	15.0
	wheat			0.996	98.3 (14.7)	108.5 (5.6)	20.8
	lettuce			0.998	83.8 (10.0)	76.2 (2.2)	20.4
	pepper			0.998	75.3 (4.1)	87.2 (3.5)	14.6
DDD pp’	apple	0.012–1.16	0.004 /0.012	0.992	90.4 (12.1)	109.0 (2.9)	17.6
	soil			0.992	95.6 (10.5)	79.2 (9.0)	24.0
	wheat			0.990	108.0 (10.1)	86.4 (4.7)	16.5
	lettuce			1.000	88.7 (7.3)	96.1 (6.0)	15.4
	pepper			0.999	78.2 (3.2)	97.7 (2.8)	10.7
DDE pp’	apple	0.010–1.00	0.003 /0.010	0.976	116.4 (7.8)	108.9 (6.9)	15.3
	soil			0.994	89.1 (15.3)	101.0 (3.6)	21.2
	wheat			0.993	85.0 (13.1)	101.9 (4.2)	20.4
	lettuce			0.999	85.8 (5.9)	93.3 (5.9)	14.7
	pepper			0.989	81.0 (6.2)	93.3 (7.1)	17.4
DDT op’	apple	0.005–0.52	0.0002 /0.005	0.994	77.5 (5.4)	102.0 (13.3)	22.7
	soil			0.991	91.7 (6.4)	96.3 (2.2)	10.3
	wheat			0.991	100.8 (6.3)	93.8 (4.2)	11.4
	lettuce			0.990	86.4 (5.0)	88.0 (6.10	15.0
	pepper			0.994	71.9 (2.4)	111.0 (3.2)	13.3
Deltamethrin	apple	0.010–1.00	0.004 /0.010	0.986	86.7 (3.8)	92.3 (2.7)	9.8
	soil			1.000	105.9 (9.3)	88.9 (3.8)	14.5
	wheat			0.997	110.3 (10.4)	103.9 (2.1)	12.5
	lettuce			0.992	96.3 (5.1)	83.4 (7.2)	15.5
	pepper			0.993	85.2 (5.0)	79.2 (7.6)	18.8
Dieldrin	apple	0.005–0.53	0.0002 /0.005	0.994	84.9 (4.9)	109.7 (5.4)	13.1
	soil			0.994	106.1 (14.0)	84.4 (7.4)	23.3
	wheat			0.986	104.7 (12.6)	94.8 (8.2)	21.1
	lettuce			0.998	90.5 (3.0)	88.6 (8.4)	14.6
	pepper			0.992	103.8 (6.9)	89.0 (4.5)	13.0
Diflufenican	apple	0.010–1.00	0.003 /0.010	1.000	89.2 (13.7)	106.4 (1.9)	18.5
	soil			0.995	89.7 (10.4)	113.8 (5.2)	18.3
	wheat			0.993	91.9 (11.7)	108.1 (4.0)	17.6
	lettuce			0.999	79.9 (2.8)	95.2 (2.2)	9.8
	pepper			1.000	85.8 (5.6)	95.1 (2.4)	10.8
Dimoxystrobin	apple	0.010–1.00	0.003 /0.010	0.996	71.9 (6.0)	91.0 (6.8)	19.7
	soil			0.997	101.0 (6.6)	112.6 (1.9)	10.8
	wheat			0.993	75.6 (2.7)	106.0 (6.5)	14.5
	lettuce			1.000	103.2 (6.4)	90.3 (4.8)	12.5
	pepper			0.999	91.1 (9.7)	87.0 (4.8)	17.8
Epoxiconazole	apple	0.010–1.00	0.003 /0.010	0.993	93.0 (5.2)	106.4 (3.5)	10.0
	soil			0.994	95.2 (6.5)	89.8 (7.0)	15.5
	wheat			0.994	96.8 (6.3)	87.2 (5.8)	14.3
	lettuce			0.997	84.4 (5.6)	82.8 (7.9)	18.9
	pepper			0.997	91.0 (10.7)	82.3 (7.0)	22.1
Esfenvalerate	apple	0.010–1.00	0.003 /0.010	0.998	105.2 (4.8)	101.5 (5.70	10.7
	soil			0.997	98.0 (4.9)	96.2 (2.2)	8.0
	wheat			0.997	95.8 (10.7)	91.5 (8.8)	21.3
	lettuce			0.998	91.3 (3.8)	103.4 (3.1)	8.3
	pepper			0.996	89.4 (5.2)	83.5 (2.5)	12.4
Etoxazol	apple	0.009–0.89	0.003 /0.009	0.988	101.5 (8.8)	87.1 (7.3)	18.0
	soil			0.993	107.9 (15.1)	79.5 (2.5)	21.0
	wheat			0.998	102.6 (5.6)	83.9 (6.3)	14.6
	lettuce			0.987	95.3 (3.8)	105.5 (5.3)	9.8
	pepper			0.995	82.6 (5.2)	103.5 (6.2)	14.3
Fenazaquin	apple	0.010–1.00	0.003 /0.010	0.999	74.7 (2.0)	87.8 (5.1)	14.8
	soil			0.998	115.9 (5.0)	108.0 (5.0)	11.7
	wheat			0.997	112.6 (10.1)	115.9 (6.8)	17.3
	lettuce			0.999	87.6 (5.3)	87.8 (3.0)	12.1
	pepper			0.998	83.5 (4.6)	92.6 (3.4)	11.8
Fenbuconazole	apple	0.010–1.00	0.003 /0.010	0.996	98.3 (4.5)	96.6 (3.3)	8.5
	soil			0.996	88.8 (0.7)	109.1 (5.6)	9.4
	wheat			0.986	105.9 (12.3)	81.7 (4.2)	19.2
	lettuce			0.997	76.0 (4.6)	77.4 (3.0)	17.0
	pepper			0.998	79.5 (8.0)	82.4 (1.9)	17.5
Fenoxycarb	apple	0.010–1.00	0.003 /0.010	0.997	107.5 (9.0)	102.2 (2.4)	11.4
	soil			0.993	84.2 (7.5)	85.6 (8.3)	20.7
	wheat			0.996	78.8 (2.2)	100.7 (12.5)	19.3
	lettuce			0.998	83.5 (3.3)	105.5 (1.5)	8.7
	pepper			0.996	99.4 (3.5)	94.7 (3.3)	7.7
Fenvalerate	apple	0.010–1.00	0.003 /0.010	0.997	104.5 (9.1)	97.3 (2.3)	11.7
	soil			0.991	88.3 (11.2)	109.1 (5.6)	19.1
	wheat			0.997	110.6 (4.6)	117.0 (4.4)	11.6
	lettuce			1.000	89.4 (1.7)	106.2 (5.4)	9.3
	pepper			0.997	106.1 (5.9)	91.8 (4.2)	11.2
Fluazifop-p-butyl	apple	0.010–1.00	0.003 /0.010	0.999	77.7 (6.3)	76.5 (3.7)	18.8
	soil			0.997	74.4 (2.0)	95.9 (3.4)	11.9
	wheat			0.991	94.1 (5.3)	94.5 (3.8)	10.5
	lettuce			0.999	88.3 (9.2)	90.1 (3.9)	16.4
	pepper			1.000	96.5 (3.5)	94.6 (4.4)	9.0
Fluquinconazole	apple	0.010–1.00	0.003 /0.010	0.972	95.6 (5.6)	77.1 (6.7)	17.0
	soil			0.995	80.8 (5.9)	82.8 (7.9)	20.0
	wheat			0.992	84.7 (5.6)	112.4 (5.9)	14.5
	lettuce			0.997	78.2 (8.4)	79.7 (2.8)	19.5
	pepper			0.998	86.7 (7.4)	90.4 (4.2)	15.0
Fludioxonil	apple	0.011–1.06	0.004 /0.011	0.933	86.8 (10.0)	89.8 (0.7)	15.4
	soil			0.990	106.7 (8.1)	100.5 (5.3)	13.4
	wheat			0.955	90.5 (13.5)	87.5 (11.9)	29.3
	lettuce			0.997	91.1 (6.3)	88.2 (2.3)	11.9
	pepper			0.995	81.4 (4.3)	78.4 (9.4)	21.2
Flufenacet	apple	0.010–1.00	0.003 /0.010	0.992	112.5 (6.3)	92.7 (4.7)	12.4
	soil			0.994	80.3 (12.8)	86.0 (2.8)	22.3
	wheat			0.996	82.9 (2.5)	107.0 (9.0)	14.6
	lettuce			0.999	94.4 (2.0)	104.7 (3.8)	6.9
	pepper			0.999	87.3 (6.3)	97.2 (4.4)	13.0
Fluopicolide	apple	0.010–1.00	0.003 /0.010	0.998	72.9 (3.6)	101.8 (5.6)	14.9
	soil			0.999	95.9 (4.1)	105.8 (1.4)	7.1
	wheat			0.997	95.3 (14.2)	102.5 (1.9)	17.3
	lettuce			0.998	87.5 (10.2)	82.6 (4.6)	19.8
	pepper			0.999	86.5 (6.4)	93.1 (4.6)	13.8
Fluopyram	apple	0.010–1.00	0.003 /0.010	0.991	85.9 (8.5)	100.3 (11.8)	22.6
	soil			0.996	83.2 (1.5)	98.1 (12.1)	17.7
	wheat			0.991	107.5 (1.7)	105.0 (5.9)	8.8
	lettuce			0.999	87.7 (6.0)	89.1 (1.9)	11.8
	pepper			1.000	84.1 (8.8)	96.0 (3.7)	15.7
Flurochloridone	apple	0.010–1.00	0.003 /0.010	0.991	104.7 (5.5)	98.6 (13.9)	19.7
	soil			0.990	103.1 (3.6)	95.5 (15.4)	20.0
	wheat			0.993	97.2 (18.2)	102.6 (8.9)	27.5
	lettuce			0.999	83.1 (7.6)	87.4 (4.5)	16.9
	pepper			0.999	74.9 (5.8)	95.9 (3.9)	15.1
Flutriafol	apple	0.010–1.00	0.003 /0.010	0.997	87.7 (7.2)	74.8 (3.0)	17.4
	soil			0.990	76.0 (5.7)	82.9 (8.4)	21.6
	wheat			0.995	108.4 (7.2)	101.9 (9.9)	16.9
	lettuce			0.998	92.9 (4.1)	72.2 (0.2)	13.1
	pepper			0.998	83.2 (1.9)	72.3 (1.8)	13.9
Folpet	apple	0.012–1.22	0.004 /0.012	0.988	75.1 (3.9)	92.1 (4.2)	14.2
	soil			0.987	89.3 (3.6)	70.6 (0.6)	13.8
	wheat			0.990	102.3 (11.6)	81.6 (4.4)	19.1
	lettuce			0.998	93.9 (3.2)	74.0 (3.4)	12.9
	pepper			0.998	88.0 (2.8)	78.7 (6.9)	15.6
HCH-alfa	apple	0.009–0.94	0.003 /0.009	0.999	95.0 (5.1)	99.0 (13.1)	18.9
	soil			0.999	85.3 (9.7)	94.3 (4.2)	17.1
	wheat			0.991	102.9 (13.9)	92.6 (9.5)	24.1
	lettuce			0.999	85.1 (1.9)	78.9 (2.1)	11.7
	pepper			1.000	85.2 (8.9)	102.0 (0.3)	12.6
HCH-beta	apple	0.010–0.98	0.003 /0.010	0.997	98.6 (8.3)	105.7 (10.8)	18.9
	soil			0.996	84.7 (11.8)	94.0 (14.2)	29.9
	wheat			0.993	87.8 (5.7)	103.4 (9.9)	17.1
	lettuce			0.998	81.7 (7.5)	100.6 (2.4)	13.3
	pepper			1.000	86.4 (11.2)	97.0 (3.5)	17.4
HCH-gamma	apple	0.011–1.10	0.004 /0.011	0.998	90.8 (3.9)	76.7 (5.3)	14.9
	soil			0.999	90.1 (9.9)	96.4 (1.4)	13.6
	wheat			0.992	110.5 (10.5)	94.6 (6.3)	17.0
	lettuce			0.999	89.3 (7.8)	79.3 (2.4)	16.2
	pepper			0.998	87.5 (6.1)	104.0 (3.8)	11.9
HCB	apple	0.012–1.24	0.004 /0.012	0.990	118.5 (1.3)	99.3 (8.7)	14.4
	soil			0.988	85.5 (8.3)	90.8 (6.0)	17.8
	wheat			0.992	105.9 (3.5)	78.3 (5.0)	12.9
	lettuce			1.000	97.5 (9.3)	72.9 (1.1)	17.7
	pepper			1.000	90.4 (6.5)	102.1 (1.2)	9.6
Hexythiazox	apple	0.010–1.00	0.003 /0.010	0.993	96.1 (3.0)	79.3 (6.7)	13.9
	soil			0.999	92.2 (3.8)	90.6 (7.1)	13.2
	wheat			0.992	95.4 (13.8)	88.7 (6.1)	22.3
	lettuce			0.986	86.0 (7.4)	101.5 (6.6)	16.2
	pepper			0.997	94.7 (4.6)	93.2 (6.3)	12.3
Heptachlor	apple	0.010–0.096	0.003 /0.010	0.990	118.5 (1.3)	99.3 (8.7)	14.4
	soil			0.990	85.5 (8.3)	90.8 (6.0)	17.8
	wheat			0.989	105.9 (3.5)	78.3 (5.0)	12.9
	lettuce			0.999	97.5 (9.3)	72.9 (1.1)	17.7
	pepper			0.999	90.4 (6.5)	102.1 (1.2)	9.6
Imazalil	apple	0.010–1.00	0.003 /0.010	0.995	102.1 (2.0)	76.2 (6.0)	12.9
	soil			0.997	92.2 (7.9)	73.5 (3.0)	17.7
	wheat			0.992	92.8 (10.2)	85.3 (11.6)	25.6
	lettuce			0.998	92.4 (3.7)	73.9 (3.1)	13.4
	pepper			0.997	93.0 (1.7)	74.6 (1.3)	10.6
Imibenconazole	apple	0.008–0.80	0.003 /0.008	0.942	85.5 (2.5)	96.0 (3.1)	8.8
	soil			0.991	93.1 (10.1)	113.2 (1.9)	15.4
	wheat			0.997	90.3 (12.9)	83.7 (11.0)	28.7
	lettuce			0.987	81.7 (3.5)	82.0 (5.1)	15.1
	pepper			0.991	85.1 (8.7)	91.1 (3.4)	15.8
Indoxakarb	apple	0.010–1.00	0.003 /0.010	0.990	110.2 (6.6)	74.9 (5.0)	16.7
	soil			0.987	104.1 (12.0)	96.9 (1.4)	13.7
	wheat			0.990	98.1 (7.6)	87.2 (9.5)	19.4
	lettuce			0.999	84.2 (6.3)	88.8 (5.5)	16.0
	pepper			0.996	85.4 (4.0)	99.0 (9.5)	16.1
Iprovalicarb	apple	0.010–1.04	0.003 /0.010	0.997	92.1 (12.2)	79.8 (5.0)	22.2
	soil			0.992	98.1 (3.7)	91.6 (1.0)	6.9
	wheat			0.988	93.8 (10.1)	102.2 (9.5)	20.4
	lettuce			0.988	86.4 (8.7)	91.0 (6.2)	18.3
	pepper			0.999	92.0 (3.6)	98.5 (4.7)	9.6
Kresoxim-methyl	apple	0.012–1.23	0.004 /0.012	0.999	93.8 (3.0)	95.6 (7.9)	12.4
	soil			0.998	90.8 (12.5)	94.6 (4.1)	18.8
	wheat			0.994	106.7 (8.0)	88.0 (6.9)	16.5
	lettuce			0.999	91.2 (3.6)	99.6 (1.7)	6.9
	pepper			1.000	75.9 (3.1)	97.8 (1.7)	10.4
Lambda-cyhalothrin	apple	0.010–1.00	0.003 /0.010	0.997	76.1 (5.5)	72.8 (2.1)	18.5
	soil			0.998	91.4 (2.2)	105.6 (0.6)	5.7
	wheat			0.992	76.5 (3.6)	111.8 (3.6)	13.2
	lettuce			1.000	92.0 (6.6)	99.6 (3.9)	11.7
	pepper			0.992	91.6 (5.9)	73.1 (2.4)	15.4
Malathion	apple	0.010–1.00	0.003 /0.010	0.996	99.1 (4.1)	94.5 (3.5)	8.5
	soil			0.996	87.5 (7.4)	101.5 (6.6)	15.9
	wheat			0.996	95.6 (14.7)	111.3 (6.7)	22.4
	lettuce			1.000	88.0 (3.8)	105.9 (3.5)	9.6
	pepper			0.998	80.4 (4.9)	91.8 (3.5)	13.0
Mepanipirym	apple	0.010–1.00	0.003 /0.010	0.996	70.3 (0.4)	102.4 (4.8)	13.6
	soil			1.000	92.8 (6.6)	110.0 (8.5)	15.8
	wheat			0.998	96.8 (6.3)	112.2 (5.7)	13.0
	lettuce			0.999	82.9 (3.2)	74.0 (0.9)	14.0
	pepper			0.997	86.8 (4.9)	92.2 (3.5)	11.5
Metalaxyl	apple	0.010–1.00	0.003 /0.010	0.990	71.0 (1.4)	91.8 (3.9)	13.7
	soil			0.988	80.7 (12.2)	77.5 (4.1)	24.6
	wheat			0.993	97.7 (5.2)	107.8 (9.0)	14.2
	lettuce			0.988	99.7 (5.0)	86.4 (2.1)	9.9
	pepper			0.998	81.6 (1.3)	100.7 (8.7)	14.4
Metamitron	apple	0.010–1.00	0.003 /0.010	0.988	83.3 (6.8)	100.1 (3.8)	13.5
	soil			0.994	80.2 (10.0)	87.0 (5.8)	21.5
	wheat			0.991	82.0 (5.0)	108.0 (9.5)	17.3
	lettuce			0.994	91.9 (4.5)	86.8 (6.3)	13.8
	pepper			0.998	90.2 (11.3)	92.7 (3.9)	17.7
Metazachlor	apple	0.011–1.06	0.004 /0.011	0.999	74.3 (4.4)	88.8 (9.1)	20.3
	soil			0.999	113.9 (3.7)	111.1 (6.6)	12.1
	wheat			1.000	98.3 (7.2)	116.9 (4.8)	13.9
	lettuce			0.999	77.2 (1.6)	85.7 (2.8)	12.4
	pepper			0.999	73.9 (3.3)	91.1 (3.4)	13.5
Metconazole	apple	0.013–1.27	0.004 /0.013	0.993	88.1 (14.1)	111.4 (6.1)	22.9
	soil			0.992	101.8 (4.1)	97.8 (13.5)	18.0
	wheat			0.991	84.0 (3.5)	78.6 (6.9)	17.1
	lettuce			0.998	94.7 (5.4)	76.9 (3.8)	14.5
	pepper			0.999	95.5 (6.7)	90.2 (3.3)	12.0
Metobromuron	apple	0.010–1.00	0.003 /0.010	0.997	95.3 (6.2)	86.5 (6.3)	15.1
	soil			0.997	77.9 (5.5)	86.4 (1.4)	14.0
	wheat			0.997	97.3 (9.3)	81.1 (2.6)	16.1
	lettuce			0.993	98.4 (4.4)	87.7 (9.20	15.9
	pepper			0.998	92.1 (3.7)	104.8 (3.0)	8.1
Metrafenone	apple	0.010–1.00	0.003 /0.010	0.993	74.5 (3.8)	81.8 (10.5)	23.0
	soil			0.996	101.2 (8.8)	112.7 (6.5)	15.7
	wheat			0.990	88.1 (12.7)	93.9 (6.1)	21.7
	lettuce			0.998	87.9 (2.1)	86.2 (5.2)	11.8
	pepper			0.992	93.2 (7.8)	94.3 (8.5)	17.9
Metribuzin	apple	0.010–1.00	0.003 /0.010	0.991	99.4 (4.0)	76.4 (2.3)	11.7
	soil			0.996	99.7 (8.6)	93.8 (10.2)	19.8
	wheat			0.996	104.8 (0.7)	96.7 (15.2)	17.7
	lettuce			0.998	94.4 (12.9)	73.3 (1.8)	22.0
	pepper			0.997	100.9 (5.2)	85.8 (3.2)	11.0
Myclobutanyl	apple	0.010–1.00	0.003 /0.010	0.992	103.9 (6.6)	82.3 (14.8)	25.3
	soil			0.993	81.6 (3.2)	97.4 (10.0)	17.1
	wheat			0.991	101.8 (16.8)	108.7 (8.0)	24.4
	lettuce			1.000	94.0 (6.2)	81.1 (5.3)	15.6
	pepper			0.998	85.0 (4.5)	89.3 (5.7)	14.1
Napropamid	apple	0.010–1.00	0.004 /0.010	0.998	108.0 (5.8)	104.6 (4.3)	10.5
	soil			0.999	78.4 (7.2)	110.3 (6.0)	17.5
	wheat			0.998	112.7 (1.6)	99.8 (3.0)	7.3
	lettuce			0.999	93.3 (8.7)	98.5 (4.2)	14.0
	pepper			1.000	85.1 (2.1)	91.4 (3.2)	9.5
Oxamyl	apple	0.010–1.02	0.003 /0.010	0.993	97.4 (4.7)	101.2 (4.7)	9.8
	soil			0.993	99.1 (6.0)	99.3 (9.4)	15.6
	wheat			0.991	89.3 (5.3)	104.5 (5.4)	12.3
	lettuce			0.990	92.3 (5.8)	95.4 (4.4)	11.7
	pepper			0.992	87.5 (4.2)	72.9 (1.7)	14.3
Oxyflurofen	apple	0.010–1.00	0.003 /0.010	0.989	93.5 (4.1)	94.1 (3.9)	9.5
	soil			0.986	85.1 (8.8)	85.4 (7.3)	20.9
	wheat			0.997	99.5 (4.1)	98.4 (7.4)	11.9
	lettuce			0.997	93.4 (3.1)	108.4 (5.1)	9.4
	pepper			0.998	105.0 (3.8)	111.3 (5.3)	9.9
Paclobutrazol	apple	0.010–1.03	0.004 /0.010	0.996	81.3 (12.6)	84.0 (11.9)	31.4
	soil			0.994	81.1 (6.8)	77.3 (5.9)	20.2
	wheat			0.992	98.9 (4.1)	87.9 (7.0)	13.0
	lettuce			0.998	94.2 (3.1)	81.9 (3.4)	10.6
	pepper			0.999	77.1 (5.9)	90.0 (5.8)	17.3
Pendimethalin	apple	0.010–1.00	0.003 /0.010	0.999	87.4 (6.0)	92.8 (9.1)	18.0
	soil			0.991	95.4 (2.9)	93.0 (5.5)	9.9
	wheat			0.998	92.4 (13.4)	114.4 (9.9)	24.4
	lettuce			0.992	75.3 (2.6)	84.3 (3.7)	14.4
	pepper			0.992	85.7 (7.7)	109.6 (3.0)	14.1
Penconazole	apple	0.010–1.00	0.003 /0.010	0.997	75.3 (6.2)	73.5 (3.1)	19.8
	soil			0.998	103.6 (5.8)	81.7 (8.4)	17.4
	wheat			0.995	92.8 (11.2)	104.2 (7.3)	19.5
	lettuce			0.999	93.7 (14.3)	80.0 (2.8)	22.2
	pepper			0.995	87.7 (6.0)	85.6 (3.2)	13.5
Penthiopyrad	apple	0.011–1.13	0.004 /0.011	0.992	78.3 (11.0)	97.3 (13.9)	29.8
	soil			0.994	103.6 (14.8)	77.3 (5.9)	24.5
	wheat			0.996	103.4 (10.2)	84.7 (7.0)	19.4
	lettuce			0.999	79.5 (5.7)	83.1 (3.9)	16.3
	pepper			0.999	78.1 (4.3)	83.8 (1.0)	13.4
Pencycuron	apple	0.010–1.00	0.003 /0.010	0.997	90.6 (4.6)	86.8 (12.8)	21.1
	soil			0.994	95.9 (3.0)	94.6 (11.0)	15.3
	wheat			0.995	107.4 (9.0)	93.2 (8.8)	18.5
	lettuce			0.994	89.9 (5.7)	82.0 (4.2)	14.5
	pepper			0.999	85.4 (9.2)	83.2 (1.9)	17.1
Phosmet	apple	0.010–1.00	0.003 /0.010	0.986	98.4 (4.5)	86.1 (6.5)	13.4
	soil			0.990	87.5 (10.6)	76.6 (6.1)	23.2
	wheat			0.991	85.5 (4.3)	83.3 (1.4)	11.9
	lettuce			0.998	88.8 (5.6)	103.9 (6.2)	13.4
	pepper			0.992	111.9 (2.7)	91.4 (6.5)	12.0
Pirimiphos-methyl	apple	0.010–1.00	0.005 /0.010	0.996	74.8 (4.4)	113.5 (3.3)	14.4
	soil			0.995	84.8 (3.9)	87.9 (8.5)	16.9
	wheat			0.998	107.2 (11.2)	89.2 (6.5)	18.8
	lettuce			1.000	89.7 (3.0)	99.7 (7.4)	12.2
	pepper			0.999	88.6 (6.6)	88.1 (5.4)	15.4
Pirimicarb	apple	0.010–1.04	0.003 /0.010	1.000	78.1 (4.7)	93.8 (6.2)	15.7
	soil			0.997	102.5 (7.1)	111.1 (5.8)	13.3
	wheat			0.998	88.2 (12.9)	115.2 (7.3)	22.8
	lettuce			1.000	85.6 (5.0)	82.0 (3.0)	13.7
	pepper			0.999	91.8 (9.1)	92.8 (4.0)	15.1
Prochloraz	apple	0.010–1.00	0.003 /0.010	0.989	98.7 (2.5)	89.7 (2.0)	6.7
	soil			0.997	74.7 (1.5)	93.9 (2.4)	10.9
	wheat			0.990	98.5 (9.4)	108.9 (1.6)	12.8
	lettuce			0.990	86.4 (4.5)	104.1 (7.4)	13.9
	pepper			0.985	91.6 (4.6)	75.3 (4.5)	15.1
Propaquizafop	apple	0.010–1.00	0.004 /0.010	0.990	100.2 (6.3)	80.2 (5.9)	15.8
	soil			0.997	115.3 (1.1)	79.1 (3.6)	12.3
	wheat			0.988	107.6 (9.9)	103.0 (1.2)	11.4
	lettuce			0.999	96.1 (9.2)	93.8 (8.2)	18.7
	pepper			0.994	108.1 (3.3)	92.3 (4.8)	9.8
Propyzamide	apple	0.010–1.00	0.003 /0.010	0.946	73.3 (3.1)	91.1 (6.1)	16.1
	soil			0.991	84 (2.5)	106 (3.9)	10.0
	wheat			0.994	88.2 (4.7)	104.6 (9.8)	16.0
	lettuce			0.998	80.1 (3.8)	89.1 (7.3)	16.4
	pepper			0.996	79.1 (7.2)	84.2 (7.2)	20.7
Pyraclostrobin	apple	0.010–1.00	0.003 /0.010	0.996	82.9 (8.1)	81.7 (5.0)	19.2
	soil			0.990	103.5 (7.7)	92.5 (5.4)	13.9
	wheat			0.995	96.4 (5.0)	75.0 (6.8)	17.1
	lettuce			0.990	101.7 (4.8)	81.2 (6.1)	14.2
	pepper			0.998	82.5 (6.1)	90.7 (5.9)	16.2
Pyridaben	apple	0.010–1.00	0.003 /0.010	0.993	91.6 (6.4)	70.1 (0.5)	16.2
	soil			0.997	111.1 (8.4)	113.9 (5.7)	14.8
	wheat			0.995	89.4 (5.9)	90.2 (1.5)	10.9
	lettuce			0.999	84.8 (10.7)	97.4 (6.1)	19.9
	pepper			0.996	79.7 (8.6)	103.5 (5.6)	17.9
Pyrimethanil	apple	0.010–1.00	0.003 /0.010	0.993	96.2 (1.4)	107.4 (7.4)	9.5
	soil			0.993	85.1 (4.3)	95.2 (14.7)	22.3
	wheat			1.000	91.1 (3.4)	95.8 (4.9)	10.1
	lettuce			1.000	79.7 (5.2)	86.7 (1.9)	13.4
	pepper			1.000	85.3 (5.3)	95.2 (1.2)	9.8
Pyriproxyfen	apple	0.010–1.00	0.004 /0.010	0.999	92.6 (8.5)	74.8 (5.4)	19.9
	soil			0.988	89.0 (12.6)	84.2 (12.0)	29.6
	wheat			0.996	96.7 (5.1)	118.3 (2.8)	11.4
	lettuce			0.994	83.7 (9.7)	90.1 (7.5)	21.5
	pepper			0.999	90.7 (5.2)	100.3 (6.9)	13.4
Quizalofop-P-ethyl	apple	0.010–1.00	0.003 /0.010	0.998	98.5 (6.2)	102.4 (3.3)	10.0
	soil			0.994	96.3 (4.8)	100.9 (2.7)	8.1
	wheat			0.996	107.9 (5.2)	106.0 (4.7)	10.4
	lettuce			0.998	84.2 (3.1)	100.8 (2.7)	8.9
	pepper			0.998	93.9 (7.7)	95.9 (4.6)	13.6
Tau-fluvanilate	apple	0.010–1.00	0.003 /0.010	0.996	90.5 (6.7)	93.1 (5.3)	14.1
	soil			0.997	93.5 (13.0)	87.6 (8.0)	24.0
	wheat			0.997	99.9 (5.0)	99.9 (5.1)	10.4
	lettuce			0.998	87.8 (3.7)	105.1 (4.2)	10.1
	pepper			0.990	94.7 (8.50	105.7 (5.4)	14.7
Tebuconazole	apple	0.010–1.00	0.003 /0.010	0.996	82.7 (6.6)	93.6 (4.5)	14.8
	soil			0.997	104.8 (3.7)	96.1 (8.5)	12.9
	wheat			0.996	90.0 (11.6)	110.5 (7.3)	20.6
	lettuce			0.998	81.5 (9.0)	78.3 (4.2)	20.6
	pepper			1.000	83.5 (9.1)	86.4 (5.5)	19.6
Terbuthylazine	apple	0.010–1.00	0.004 /0.010	0.998	81.4 (8.2)	96.1 (3.2)	15.2
	soil			0.998	91.0 (8.1)	110.1 (8.3)	17.6
	wheat			0.995	83.4 (4.4)	91.9 (3.2)	11.6
	lettuce			0.999	96.1 (3.8)	88.8 (3.5)	9.5
	pepper			0.999	95.5 (3.8)	91.0 (5.2)	10.8
Tetraconazole	apple	0.010–1.00	0.003 /0.010	0.993	79.7 (4.2)	104.7 (3.9)	12.1
	soil			0.999	88.7 (5.1)	94.1 (2.7)	10.2
	wheat			0.999	103.0 (4.8)	110.0 (3.3)	9.2
	lettuce			0.998	96.4 (1.7)	99.3 (2.8)	5.5
	pepper			0.999	105.1 (1.6)	97.6 (7.5)	10.2
Tolclofos methyl	apple	0.010–1.00	0.003 /0.010	1.000	83.5 (4.2)	111.2 (2.8)	11.3
	soil			1.000	80.5 (8.6)	116.7 (5.0)	18.7
	wheat			0.998	106.4 (4.5)	105.3 (8.7)	13.3
	lettuce			1.000	99.4 (1.5)	98.1 (1.8)	4.2
	pepper			1.000	89.2 (8.5)	97.1 (1.9)	12.5
Trifloxystrobin	apple	0.010–1.00	0.003 /0.010	0.999	86.4 (5.6)	90.6 (8.1)	17.1
	soil			0.993	98.4 (7.3)	114.3 (6.2)	14.5
	wheat			0.995	83.1 (13.3)	119.3 (3.3)	23.0
	lettuce			0.998	73.9 (1.8)	92.8 (1.2)	10.7
	pepper			0.997	90.2 (8.2)	91.0 (7.3)	18.1
Triflumizole	apple	0.010–1.00	0.003 /0.010	0.993	106.2 (6.6)	101.5 (4.7)	11.4
	soil			0.995	109.9 (5.3)	97.9 (7.1)	13.1
	wheat			0.999	99.3 (4.6)	107.0 (6.9)	11.7
	lettuce			0.999	102.8 (5.4)	86.4 (11.5)	19.4
	pepper			0.995	101.0 (4.2)	97.4 (8.1)	12.7
Triticonazole	apple	0.010–1.00	0.004 /0.010	0.999	112.3 (3.4)	111.4 (4.4)	10.1
	soil			0.998	80.5 (3.6)	81.0 (4.9)	15.5
	wheat			0.998	102.3 (2.3)	107.5 (1.1)	5.3
	lettuce			0.998	89.0 (5.0)	92.8 (8.4)	15.9
	pepper			0.994	108.7 (4.8)	85.6 (5.8)	13.2

**Table 4:** Validation parameters for PAH determinations (linearity range, calibration curve and coefficient of determination, recovery, standard deviation from recoveries (RSD), measurement uncertainty (U)).

Compound	Matrix Type	Linearity Range [mg/kg]	LOD /LOQ (mg/kg)	R^2^	Recovery % (RSD, n = 5)		U (k = 2) [%]
					LOQ	1000 × LOQ	
Acenaphtylene	apple	0.001–1.00	0.0003 /0.001	0.988	99.6 (7.8)	97.7 (0.4)	9.4
	soil			1.000	74.5 (1.6)	98.9 (0.2)	9.0
	wheat			1.000	88.4 (1.9)	101.0 (1.7)	6.2
	lettuce			0.992	98.1 (4.5)	99.3 (6.9)	11.8
	pepper			1.000	79.0 (6.2)	103.9 (4.6)	14.7
Anthracene	apple	0.001–1.00	0.0003 /0.001	0.996	105.4 (3.0)	97.0 (2.3)	6.2
	soil			1.000	82.7 (4.8)	97.8 (0.7)	9.3
	wheat			1.000	98.5 (3.1)	102.9 (3.2)	6.8
	lettuce			0.997	106.2 (2.5)	100.7 (7.9)	11.1
	pepper			1.000	99.4 (5.6)	99.3 (5.9)	11.8
Benzo[a]anthracene	apple	0.001–1.00	0.0003 /0.001	1.000	90.0 (7.5)	99.6 (1.1)	10.5
	soil			1.000	72.0 (1.3)	96.0 (2.5)	11.4
	wheat			1.000	82.2 (0.4)	103.3 (1.3)	7.3
	lettuce			1.000	92.6 (4.1)	97.1 (10.7)	16.1
	pepper			1.000	104.1 (5.2)	98.4 (4.5)	9.9
Benzo[a]pyrene	apple	0.001–1.00	0.0003 /0.001	1.000	79.3 (2.5)	97.9 (4.9)	12.1
	soil			0.999	71.6 (1.9)	90.9 (1.2)	11.9
	wheat			1.000	96.9 (1.4)	102.0 (1.9)	4.3
	lettuce			1.000	101.8 (3.6)	97.8 (8.4)	12.4
	pepper			1.000	89.9 (4.0)	94.9 (4.7)	10.7
Benzo[b]fluoranthrene	apple	0.001–1.00	0.0003 /0.001	0.999	88.9 (1.0)	100.1 (3.2)	7.0
	soil			1.000	70.7 (0.9)	91.7 (2.1)	12.1
	wheat			1.000	96.6 (3.5)	104.0 (1.9)	6.4
	lettuce			1.000	107.3 (3.0)	98.0 (8.3)	12.3
	pepper			1.000	102.6 (4.0)	97.0 (4.8)	9.3
Benzo[k]fluoranthrene	apple	0.001–1.00	0.0003 /0.001	1.000	95.4 (5.0)	94.8 (2.6)	9.8
	soil			0.999	72.7 (1.8)	93.1 (2.5)	11.8
	wheat			1.000	93.5 (1.3)	100.9 (1.4)	4.5
	lettuce			0.999	100.0 (2.1)	96.4 (7.3)	10.1
	pepper			1.000	88.1 (2.2)	97.9 (4.9)	9.6
Benzo[ghi]perylene	apple	0.001–1.00	0.0003 /0.001	0.999	114.6 (3.3)	94.7 (8.4)	14.3
	soil			1.000	72.0 (1.6)	97.5 (5.9)	14.7
	wheat			1.000	93.7 (0.9)	96.2 (1.8)	4.8
	lettuce			1.000	103.0 (1.8)	95.3 (7.7)	10.6
	pepper			1.000	106.8 (4.5)	92.1 (4.2)	10.0
Chrysene	apple	0.001–1.00	0.0003 /0.001	0.999	108.2 (1.4)	100.5 (0.5)	4.2
	soil			0.999	71.1 (1.6)	97.5 (3.3)	12.4
	wheat			1.000	108.6 (0.2)	101.3 (1.3)	4.5
	lettuce			1.000	105.4 (3.0)	93.1 (11.2)	15.6
	pepper			1.000	110.1 (3.8)	92.6 (3.5)	9.2
Dibenzo[a,h]anthracene	apple	0.001–1.00	0.0003 /0.001	1.000	97.7 (1.4)	97.7 (5.1)	7.4
	soil			1.000	82.2 (1.7)	96.9 (7.3)	13.3
	wheat			1.000	94.7 (2.6)	100.6 (2.5)	6.1
	lettuce			1.000	102.1 (1.2)	97.9 (7.0)	9.1
	pepper			1.000	115.1 (2.2)	89.5 (6.7)	13.1
Fluorene	apple	0.001–1.00	0.0003 /0.001	0.994	90.3 (0.6)	98.1 (0.8)	8.8
	soil			1.000	91.7 (2.2)	98.8 (0.3)	4.8
	wheat			1.000	88.9 (2.6)	102.2 (2.2)	7.0
	lettuce			0.996	111.3 (4.1)	100.4 (7.1)	12.3
	pepper			1.000	75.7 (2.4)	102.6 (3.9)	11.9
Indeno[1,2,3-cd]pyrene	apple	0.001–1.00	0.0003 /0.001	0.990	96.4 (8.8)	90.2 (8.1)	18.8
	soil			0.999	84.1 (3.0)	88.0 (4.3)	12.0
	wheat			1.000	91.0 (4.1)	98.1 (1.6)	7.4
	lettuce			1.000	105.7 (2.0)	92.8 (8.5)	12.2
	pepper			1.000	109.1 (4.9)	99.5 (4.1)	9.7
Phenanthrene	apple	0.001–1.00	0.0003 /0.001	0.992	105.4 (3.0)	97.0 (2.3)	10.8
	soil			1.000	82.7 (4.8)	97.8 (0.7)	4.4
	wheat			1.000	98.5 (3.1)	102.9 (3.2)	7.3
	lettuce			0.996	106.2 (2.5)	100.7 (7.9)	16.1
	pepper			1.000	99.4 (5.6)	99.3 (5.9)	9.6
Pyrene	apple	0.001–1.00	0.0003 /0.001	0.995	97.3 (2.7)	98.9 (1.4)	5.0
	soil			1.000	81.5 (3.8)	99.5 (2.1)	9.6
	wheat			1.000	94.9 (2.9)	102.5 (2.1)	6.0
	lettuce			0.996	106.7 (8.4)	102.5 (10.6)	18.6
	pepper			1.000	93.8 (2.9)	103.9 (4.3)	8.2

**Table 5:** Pesticide residues analysis in real plant samples.

Product	Active Substance (Category)	Concentration [mg/kg]	MRL [mg/kg]
Apple	Boscalid (F)	0.37	2
Apple	Boscalid (F)	0.157	2
	Tetraconazole (F)	0.018	0.3
Apple	Boscalid (F)	0.468	2
	Etoxazole (I)	0.066	0.07
	Tetraconazole (F)	0.019	0.3
Apple	Boscalid (F)	3.595 *	2
	Tetraconazole (F)	0.02	0.3
Apple	Boscalid (F)	2.902 *	2
Apple	Boscalid (F)	2.862 *	2
Apple	Tebconazole (F)	0.135	0.3
	Boscalid (F)	3.651 *	2
	Fluopyram (F)	0.102	0.6
	Tetraconazole (F)	0.013	0.3
Apple	Boscalid (F)	0.303	2
Lettuce	Boscalid (F)	0.262	50
	Cyprodinil (F)	0.287	15
Lettuce	Boscalid (F)	5.814	50
Lettuce	Boscalid (F)	0.027	50
	Cyprodinil (F)	0.262	15
Lettuce	Boscalid (F)	0.316	50
	Cyprodnil (F)	0.847	15
Lettuce	Boscalid (F)	0.046	50
Lettuce	Boscalid (F)	0.358	50
Wheat	Pirymifos-metyl (I)	0.096	5
Wheat	Tetraconazole (F)	0.014	0.1
Wheat	Tetraconazole (F)	0.031	0.1
Wheat	Pendimethalin (H)	0.045	0.05
Wheat	Pendimethalin (H)	0.021	0.05
	Metrafenone (F)	0.025	0.07
	Boscalid (F)	0.012	0.8
Wheat	Metrafenone (F)	0.013	0.07
Wheat	Metrafenone (F)	0.012	0.07
	Boscalid (F)	0.036	0.8
Pepper	Azoxystrobin (F)	0.013	3
Pepper	n.d.	–	–
Pepper	n.d.	–	–
Pepper	n.d.	–	–
Pepper	Fluopyram (F)	0.01	3
Pepper	Boscalid (F)	0.013	3
	Fluopyram (F)	0.011	3
Pepper	Azoxystrobin (F)	0.016	3

n.d. no detected; MRL maximum residue level, F fungicide, I insecticide, H herbicide; * violated MRL.

**Table 6:** PAHs analysis in real soil samples.

Soil Sample (Place of Collection)	PAH Compounds	Concentration [mg/kg]	Land Type	MRL [mg/kg]
1 (railroad tracks)	Anthracene	0.002	IV	20
	Benzo[a]anthracene	0.001		20
	Benzo[a]pyrene	0.001		20
	Benzo[b]fluoranthrene	0.002		20
	Benzo[ghi]perylene	0.001		20
	Benzo[k]fluoranthrene	0.002		20
	Chrysene	0.002		
	Indeno[1,2,3-cd]pyrene	0.001		20
	Phenanthrene	0.001		
	Pyrene	0.003		
2 (residential)	n.d.	–	I	–
3 (farmland)	n.d.	–	II	–
4 (power plant)	Benzo[a]anthracene	0.001	IV	20
	Benzo[a]pyrene	0.001		20
	Benzo[b]fluoranthrene	0.001		20
	Benzo[k]fluoranthrene	0.001		20
	Benzo[ghi]perylene	0.001		20
	Chrysene	0.001		20
	Indeno[1,2,3-cd]pyrene	0.001		20
	Phenanthrene	0.002		
	Pyrene	0.002		
5 (ironworks)	Anthracene	0.016	IV	20
	Benzo[a]anthracene	0.065		20
	Benzo[a]pyrene	0.059		20
	Benzo[b]fluoranthrene	0.06		20
	Benzo[ghi]perylene	0.04		20
	Benzo[k]fluoranthrene	0.049		20
	Chrysene	0.068		20
	Dibenzo[a,h]anthracene	0.008		20
	Fluorene	0.002		
	Indeno[1,2,3-cd]pyrene	0.065		20
	Phenanthrene	0.031		
	Pyrene	0.092		
6 (sewage treatment plant)	Anthracene	0.028	IV	20
	Benzo[a]anthracene	0.025		20
	Benzo[a]pyrene	0.017		20
	Benzo[b]fluoranthrene	0.017		20
	Benzo[ghi]perylene	0.009		20
	Benzo[k]fluoranthrene	0.013		20
	Chrysene	0.021		20
	Dibenzo[a,h]anthracene	0.002		20
	Fluorene	0.009		
	Indeno[1,2,3-cd]pyrene	0.015		20
	Phenanthrene	0.06		
	Pyrene	0.045		
7 (land near the San river)	Phenanthrene	0.001	I	–
	Pyrene	0.001		
8 (city center)	Anthracene	0.011	IV	20
	Benzo[a]anthracene	0.066		20
	Benzo[a]pyrene	0.086		20
	Benzo[b]fluoranthrene	0.09		20
	Benzo[ghi]perylene	0.05		20
	Benzo[k]fluoranthrene	0.065		20
	Chrysene	0.072		20
	Dibenzo[a,h]anthracene	0.014		20
	Fluorene	0.001		
	Indeno[1,2,3-cd]pyrene	0.092		20
	Phenanthrene	0.019		
	Pyrene	0.074		
9 (highway)	Anthracene	0.003	IV	20
	Benzo[a]anthracene	0.02		20
	Benzo[a]pyrene	0.024		20
	Benzo[b]fluoranthrene	0.027		20
	Benzo[ghi]perylene	0.017		20
	Benzo[k]fluoranthrene	0.022		20
	Chrysene	0.026		20
	Dibenzo[a,h]anthracene	0.003		20
	Indeno[1,2,3-cd]pyrene	0.027		20
	Phenanthrene	0.006		
	Pyrene	0.024		
10 (river)	Anthracene	0.001	I	0.2
	Benzo[a]anthracene	0.003		0.2
	Benzo[a]pyrene	0.004		0.1
	Benzo[b]fluoranthrene	0.005		0.1
	Benzo[ghi]perylene	0.002		0.1
	Benzo[k]fluoranthrene	0.003		0.1
	Chrysene	0.004		0.2
	Dibenzo[a,h]anthracene	0.001		0.1
	Indeno[1,2,3-cd]pyrene	0.003		0.2
	Phenanthrene	0.003		
	Pyrene	0.006		

Land type I: residential areas, other built-up areas, urbanized undeveloped areas or areas under development, developed agricultural land, recreation and leisure areas, land type II: arable land and areas of family allotment gardens established on land, orchards, permanent meadows, permanent pastures, land under ponds, land under ditches, land type III: forests, wooded and shrubby land, wooded and shrubby land on agricultural land, wasteland, recreational areas, ecological sites, various areas, land type IV: industrial areas, fossil land, areas of traffic routes including: roads, railway areas and other traffic-related areas, land intended for the construction of public roads or railroads [14].

## Data Availability

Available on request and with regulations.

## Supplementary Materials

The following supporting information can be downloaded at: https://www.mdpi.com/article/10.3390/molecules27072140/s1, Table S1: Multiple reactions monitoring MRM transition and retention times for pesticide residues. Table S2. Single ion monitoring SIM and retention times for PAHs. Figure S1: Chromatogram for the blank sample—soil matrix. Figure S2. Chromatogram for the fortified sample (level 0.001 mg/kg)—soil matrix, PAHs (Rt: *7.27—Acenaphtylene; 8.18—Fluorene; 9.76—Phenanthrene; 9.85—Anthracene; 12.84—Pyrene; 16.02—Benzo[a]anthracene; 16.15—Chrysene; 18.96—Benzo[b]fluoranthrene; 19.02—Benzo[k]fluoranthrene; 19.78—Benzo[a]pyrene; 22.62—Dibenzo[a,h]anthracene; 23.30—Benzo[ghi]perylene*). Figure S3. Chromatogram for the real sample—soil (sample No. 7) *(Rt: 9.75—Phenanthrene; 12.83—Pyrene)*. Figure S4. Chromatogram for the blank sample—apple matrix. Figure S5. Chromatogram for the fortified sample (level 0.001 mg/kg)—apple matrix, selected pesticides *(Rt: 9.592—Oxamyl; 17.412—Heptachlor; 17.883—Metalaxyl; 21.147—Cyprodinil; 26.082—Benalaxyl; 27.815—Diflufenican)*. Figure S6. Chromatogram for the real sample—apple (sample No. 7) *(Rt: 33.238—Boscalid)*. Figure S7. Chromatogram for the blank sample—lettuce matrix. Figure S8. Chromatogram for the fortified sample (level 1.00 mg/kg)—lettuce matrix, selected pesticides *(Rt: 12.195—Pencycuron; 13.802—Clomazone; 16.230—Pirimicarb; 21.312—Metazachlor; 23.422—Mepanipirym; 24.797—Buprofezin; 29.077—Fenazaquin; 31.980—Fenbuconazole)*. Figure S9. Chromatogram for the real sample—lettuce (sample No. 7) *(Rt: 21.081—Cyprodnil; 33.163 Boscalid).*

## Sample Availability

Samples of the compounds are not available from the authors.
