# Supplementary material for: Simultaneous Determination of Multi-Class Pesticide Residues and PAHs in Plant Material and Soil Samples Using the Optimized QuEChERS Method and Tandem Mass Spectrometry Analysis
Source: Molecules. 2022 Mar 25;27(7):2140. doi: 10.3390/molecules27072140 (PMC9000817; doi:10.3390/molecules27072140)
Supplement: Supplementary file 1 [file molecules-27-02140-s001.zip › molecules-1642593-supplementary.pdf]

Supplementary material: Figures S1 – S9.

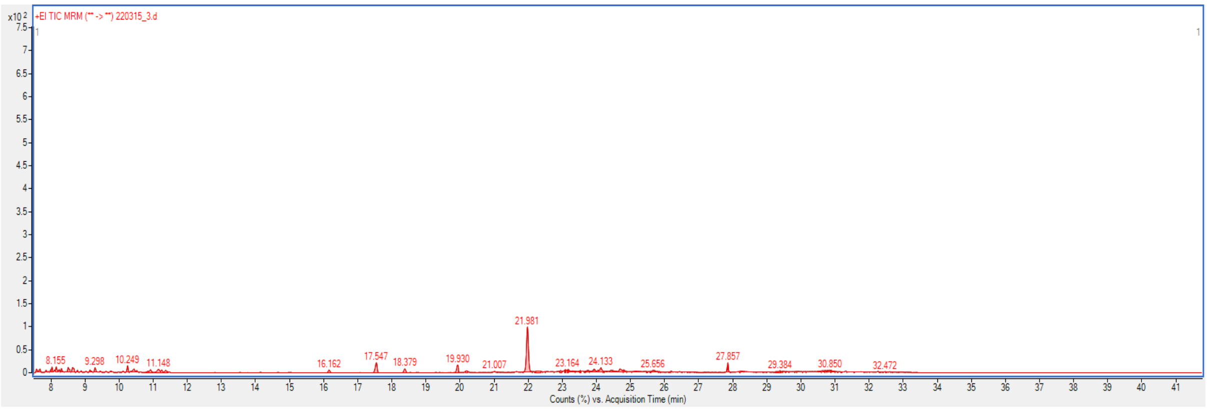

Figure S1. Chromatogram for the blank sample - soil matrix

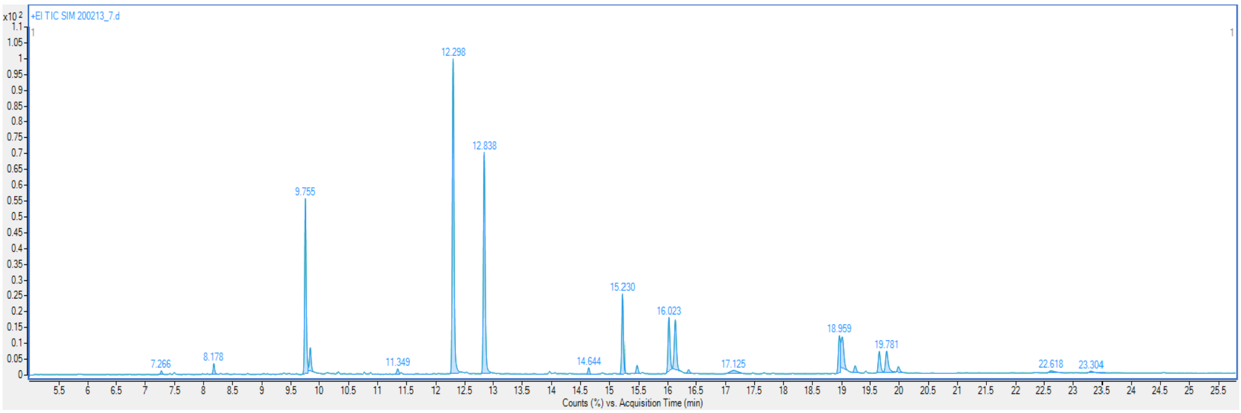

Figure S2. Chromatogram for the fortified sample (level 0.001 mg/kg) - soil matrix, PAHs *(Rt: 7.27 - Acenaphtylene; 8.18 - Fluorene; 9.76 - Phenanthrene; 9.85 - Anthracene; 12.84 - Pyrene; 16.02 - Benzo[a]anthracene; 16.15 - Chrysene; 18.96 - Benzo[b]fluoranthrene; 19.02 - Benzo[k]fluoranthrene; 19.78 - Benzo[a]pyrene; 22.62 - Dibenzo[a,h]anthracene; 23.30 - Benzo[ghi]perylene)*

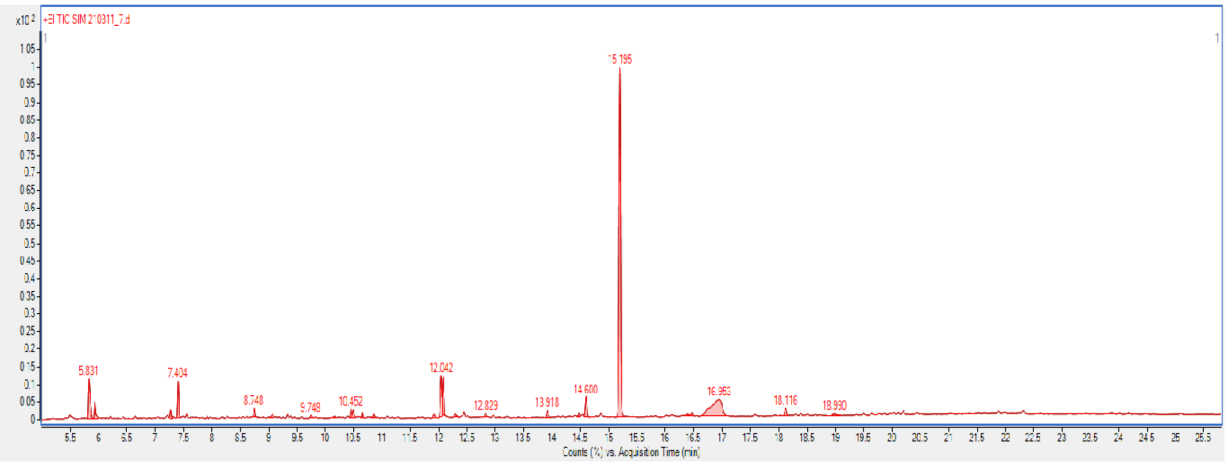

Figure S3. Chromatogram for the real sample - soil (sample No. 7) *(Rt: 9.75 - Phenanthrene; 12.83 - Pyrene)*

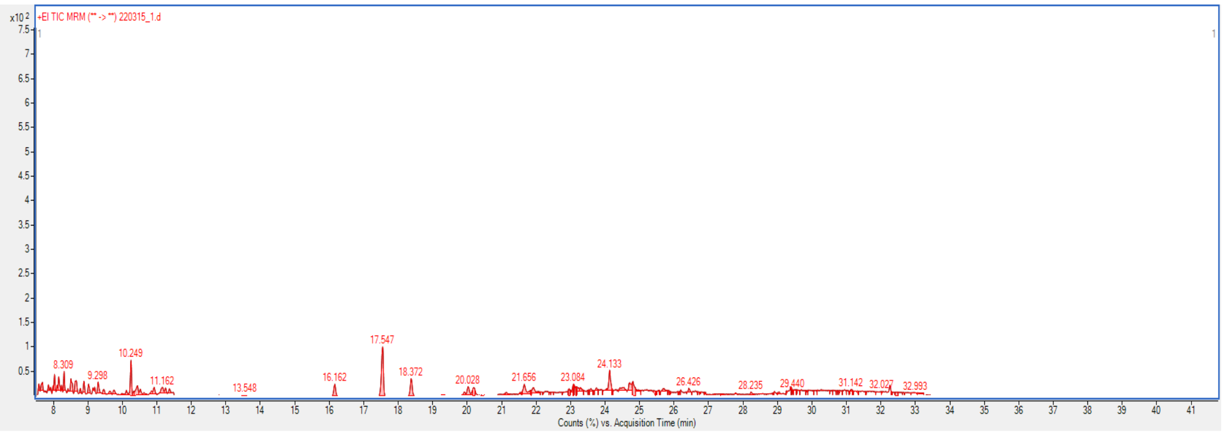

Figure S4. Chromatogram for the blank sample - apple matrix

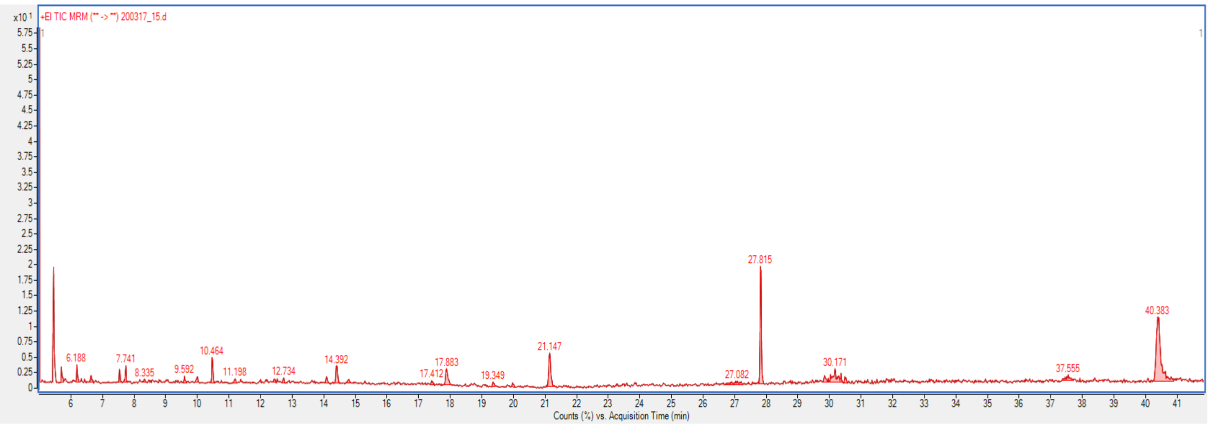

Figure S5. Chromatogram for the fortified sample (level 0.001 mg/kg) - apple matrix, selected pesticides *(Rt: 9.592 - Oxamyl; 17.412 - Heptachlor; 17.883 - Metalaxyl; 21.147 - Cyprodinil; 26.082 - Benalaxyl; 27.815 - Diflufenican)*

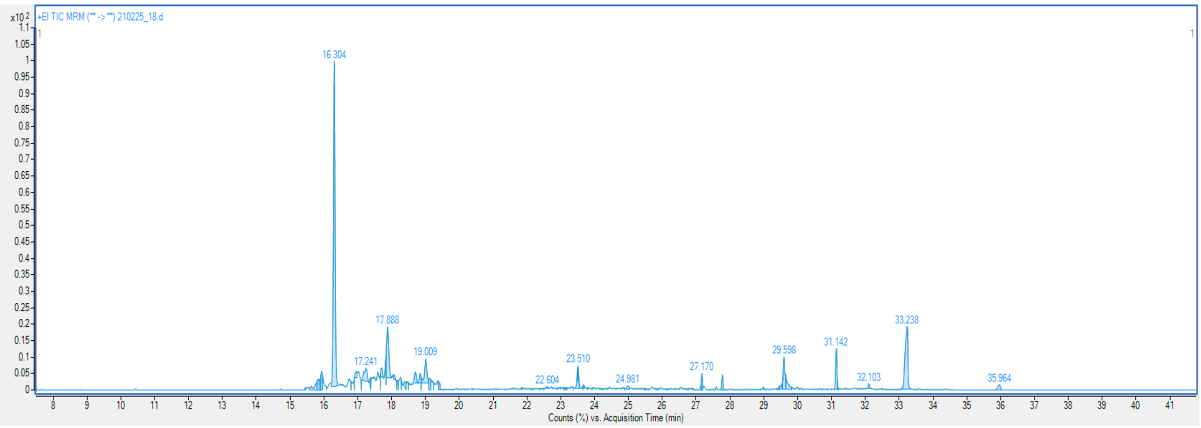

Figure S6. Chromatogram for the real sample - apple (sample No. 7) *(Rt: 33.238 - Boscalid)*

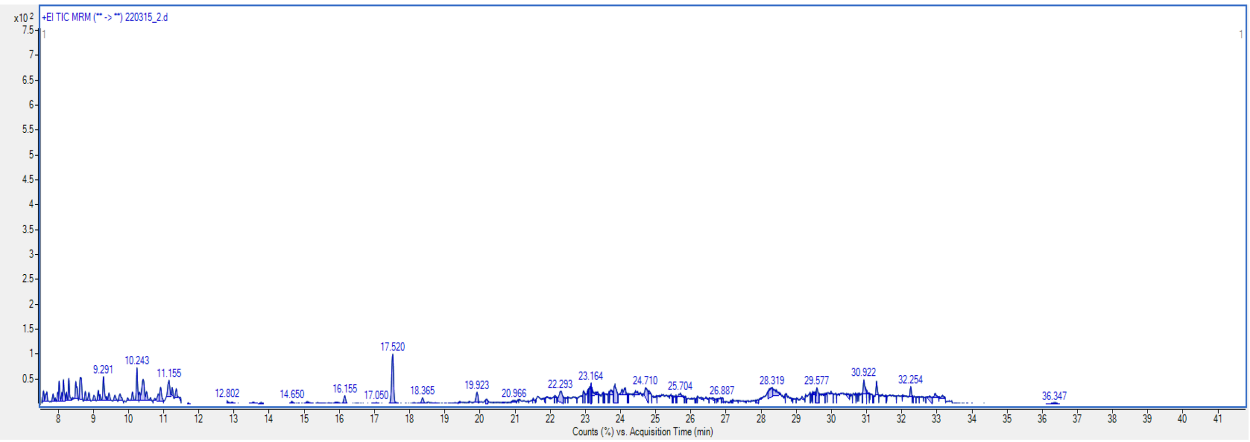

Figure S7. Chromatogram for the blank sample - lettuce matrix

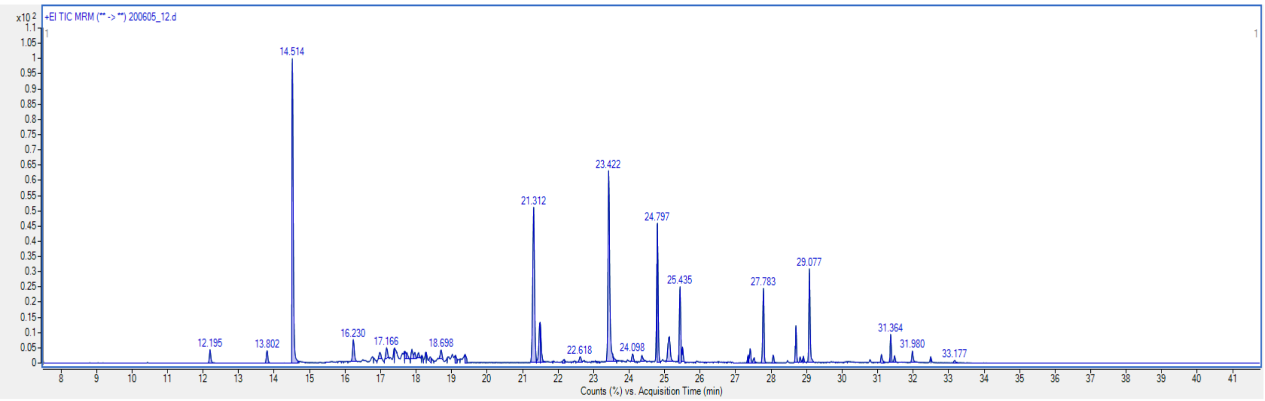

Figure S8. Chromatogram for the fortified sample (level 1.00 mg/kg) - lettuce matrix, selected pesticides *(Rt: 12.195 - Pencycuron; 13.802 - Clomazone; 16.230 - Pirimicarb; 21.312 - Metazachlor; 23.422 - Mepanipirym; 24.797 - Buprofezin; 29.077 - Fenazaquin; 31.980 - Fenbuconazole)*

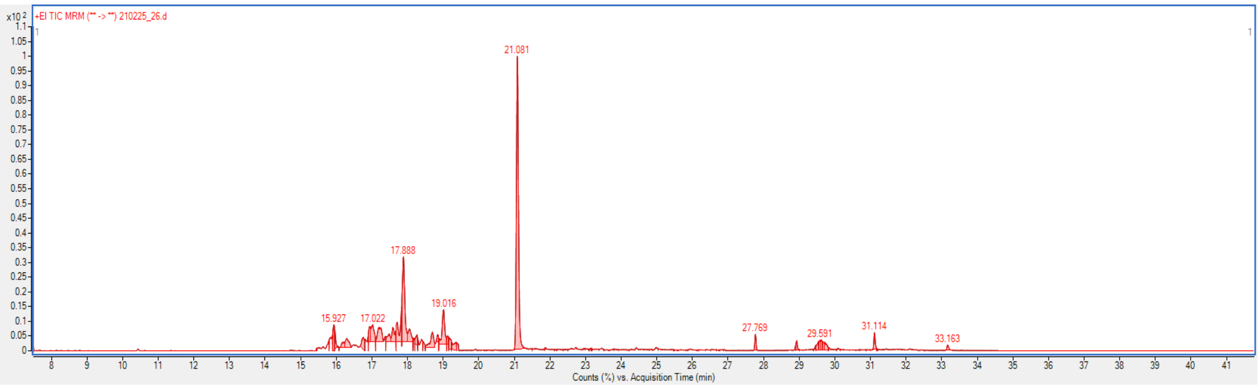

Figure S9. Chromatogram for the real sample - lettuce (sample No. 7) *(Rt: 21.081 - Cyprodnil; 33.163 Boscalid)*

Table S1. Multiple reactions monitoring MRM transition and retention times for pesticide residues.

**Retention time (min)**	**Active substance (pesticide type)**	**MRM transitions m/z (collision energy, eV)**		
		**Quantification**	**Identification (ion pairs I)**	**Identification (ion pairs II)**
9.536	Oxamyl (I)	98 > 58.1 (10)	98 > 69.1 (10)	162 > 115 (10)
11.858	Propyzamide (H)	172.8 > 145 (15)	174.9 > 146.7 (15)	172.8 > 109 (30)
12.297	Pencycuron (F)	208.1 >125 (20)	180.1 > 125 (20)	180.1 > 89.1 (30)
12.695	HCH alpha (I)	216.7 > 180.7 (5)	218.8 > 183 (5)	180.7 > 144.9 (15)
12.999	HCB (I)	283.7 > 213.9 (30)	283.7 > 248.8 (15)	288.8 > 211.8 (30)
13.833	HCH beta (I)	180.9 > 144.7 (15)	216.9 > 180.9 (5)	218.9 > 182.8 (5)
13.862	Clomazone (H)	124.8 > 89.9 (15)	204 > 107 (20)	124.8 > 98.9 (15)
14.082	HCH gamma	216.7 > 180.9 (5)	218.9 > 182.9 (5)	180.9 > 144.8 (15)
14.802	Pyrimethanil (F)	198.1 > 183 (15)	198.1 > 118 (35)	198.1 > 158 (20)
15.678	Tefluthrin (I)	177 > 127 (15)	197 > 141.1 (10)	177 > 87 (30)
16.165	Metobromuron (H)	197 > 90.1 (20)	198.9 > 90.1 (20)	198.9 > 169.9 (10)
16.286	Pirimicarb (I)	238 > 166 (10)	166 > 54.9 (20)	166 > 96 (15)
16.468	Terbuthylazine (H)	173 > 172 (5)	229.1 > 173 (5)	173 > 138 (5)
16.880	Metribuzin (H)	197.9 > 81.9 (15)	197.9 > 55.2 (30)	143.8 > 126.8 (10

17.238	Chlorpyrifos methyl (I)	125 > 47 (15)	125 > 79 (5)	285.9 > 93 (20)
17.435	Heptachlor (I)	99.8 > 58 (10)	100 > 72 (5)	198 > 126 (5)
17.458	Tolclofos methyl (F)	265 > 249.9 (15)	265 > 93 (25)	125 > 47.1 (15)
17.963	Metalaxyl (F)	220.1 > 192.1 (5)	234.1 > 146 (20)	234.1 > 174.1 (10)
18.389	Malathion (I)	127 > 99 (5)	173 > 99 (15)	158 > 124.9 (5)
18.908	Pirimiphos methyl (I)	290.1 > 125 (20)	233 > 151 (5)	233 > 125 (5)
19.843	Chlorpyrifos (I)	196.9 > 168.9 (15)	198.9 > 170.9 (15)	313.9 > 257.9 (15)
20.214	Flufenacet (H)	151.1 > 136 (10)	151.1 > 95.1 (30)	123 > 95.1 (20)
20.427	Tetraconazole (F)	171 > 136 (10)	336.1 > 218.1 (20)	336.1 > 204.1 (30)
20.471 - 20.548	Flurochloridone (H)	186.7 > 158.9 (10)	311 > 173.9 (15)	145.1 > 95.1 (15)
21.163	Cyprodinil (F)	225.1 > 224.1 (10)	224.1 > 208.1 (20)	226.2 > 225.1 (10)
21.377	Metazachlor (H)	133 > 132 (10)	132 > 117 (15)	208.9 > 132 (15)
21.548	Pendimethalin (H)	252>162.2(10)	252 > 161.1 (15)	162 > 161.1 (10)
21.800	Captan (F)	151.1 > 80.2 (5)	149 > 79.1 (10)	151.1 > 79.1 (15)
21.923	Penconazole (F)	248.1 > 192.1 (15)	248.1 > 157 (25)	159 > 89 (35)
22.160	Folpet (F)	260.2 > 130 (15)	261.9 > 130 (15)	260.2 > 231.9 (5)
22.229	Fluopyram	176.9 > 101	222.9 > 196.0 (10)	222.9 > 187.1 (10)
22.391	Zoxamide (F)	187 > 159 (15)	189 > 160.9 (15)	187 > 122.9 (30)
22.640	Hexythiazox (A)	227 > 149.1 (10)	184 > 149.1 (10)	184 > 115 (20)
22.787	Triflumizole (F)	206 > 179 (15)	206 > 186 (10)	179 > 143.9 (15)
22.888 23.580	Iprowalikarb (F)	119 > 90.9 (15)	115 > 97.9 (5)	115 > 55 (15)
23.036	Paclobutrazol (F)	235.7 > 125 (10)	125 > 89.8 (20)	235.7 > 166.9 (10)
23.500	Mepanipirym (F)	223.2 > 222.2 (10)	222.2 > 207.1 (15)	221.2 > 220.1 (15)
23.605	Flutriafol (F)	123 > 95 (15)	123 > 75 (25)	219 > 123 (15)
23.863	Napropamide (H)	128.1 > 72.1 (5)	128.1 > 100.1 (10)	271.2 > 72.1 (15)
24.125	Imazalil (F)	215 > 172.9 (5)	217 > 175 (5)	172.9 > 144.9 (15)
24.248	Dieldryna	396 > 222.9	172.9 > 145 (15)	172.9 > 141 (15)
24.375	p,p′ DDE (I)	245.9 > 176 (30)	315.9 > 245.9 (15)	317.8 > 245.9 (15)
24.666	Oxyflurofen (H)	252.1 > 196 (20)	252.1 > 146 (30)	300 > 223 (15)
24.679	o,p′ DDT (I)	235 > 165.1 (20)	236.9 > 165.1 (20)	235 > 199 (15)
24.759	Myclobutanyl (F)	179 > 125 (10)	179 > 90(30)	150 > 122.9 (15)
24.933	Buprofezin (I)	105.1 > 104 (10)	105 > 77.1 (20)	119 > 91 (15)
25.07	Bupirimate (F)	272.9 > 192.8 (5)	272.9 > 107.8 (15)	207.9 > 166.1 (10)
25.154	Kresoxim-methyl (F)	116 > 89 (15)	116 > 63.8 (30)	131.1 > 89 (30)
25.187	Cyproconazole (I)	139 > 111.1 (15)	222 > 125 (15)	222 > 82.1 (10)
25.510	Cyflufenamid (F)	118 > 90 (10)	118 > 89 (25)	188 > 88 (35)
25.554	Fluazifop-p-butyl (H)	282.2 > 91 (20)	282.2 > 238 (20)	383.2 > 282.2 (10)

25.613-25.771	Metamitron (H)	103.9 > 77.2 (15)	202.2 > 174.1 (5)	103.9 > 51.1 (35)
25.926	p,p′ DDD (I)	235 > 164.7 (20)	237 > 164.7 (20)	235 > 199 (15)
26.208	Pentiopirad	302.1 > 151.9 (15)	302.1 > 177.0 (15)	302 > 176.9 (15)
26.212	Fludioxonil (F)	248 > 153.9 (20)	248.1 > 181.9 (10)	248.1 > 127 (30)
26.889, 27.101	Prothioconazole (F)	186 > 53 (20)	186 > 117 (10)	186 > 89 (10)
26.906	Benalaxyl (F)	148.1 > 105.1 (20)	148.1 > 77 (35)	266.1 > 148.1 (5)
26.959	p.p′ DDT (I)	235 > 165 (20)	237 > 165 (20)	235 > 199 (15)
27.406	Trifloxystrobin (F)	116.2 > 89 (15)	172 > 144.8 (15)	116.2 > 63.2 (30)
27.470	Fluopicolide (F)	209 > 182 (10)	209 > 146 (20)	347 > 171.9 (20)
27.576	Tebuconazole (F)	124.9 > 89.1 (15)	250.1 > 124.9 (20)	124.9 > 99.2(20)
27.883	Diflufenican (DFF) (H)	266 > 238.1 (15)	266 > 246 (15)	394 > 266 (10)
28.152	Epoxiconazole (F)	192 > 138 (10)	192 > 111 (25)	165 > 138 (10)
28.521, 29.202	Bromuconazole (F)	173 > 144.9 (15)	173 > 108.9 (30)	174.9 > 146.9 (15)
28.739	Phosmet (I)	160 > 77.2 (20)	160 > 133 (10)	160 > 105 (15)
28.781	Dimoksystrobina (F)	116 > 89 (15)	116 > 63.1 (30)	205.1 > 116 (10)
28.880	Fenoxycarb (I)	255.1 > 185.9 (10)	185.9 > 158.1 (5)	185 > 129. (5)
28.881	Bifenazat (I)	184.1 > 156.1 (10)	199.1 > 184.1 (10)	184.1 > 169 (10)
28.882	Chlorantraniliprole (I)	278 > 249.1 (25)	278 > 215 (30)	239 >214 (20)
28.892	Acetamiprid (I)	152 > 116.1 (15)	125.9 > 90 (5)	125.9 > 98.8 (10)
29.086	Etoxazol (A)	140.9 > 113.2 (15)	140.9 > 63 (30)	204 > 175.9 (10)
29.09, 29.2	Metconazol (F)	125 > 88.8 (20)	125 > 99 (20)	138 > 69.1 (10)
29.177	Fenazaquin (A)	145.2 > 117.1 (10)	160.1 > 145.2 (5)	160.1 > 117.1 (20)
29.563	Triticonazole (F)	235.1 > 217 (5)	235.1> 182.2 (10)	217 > 165 (25)
29.586, 29.352	Acrinathrin (I)	207 > 181.2 (10)	181.2 > 152.1 (30)	289.2 > 93 (10)
29.853	Pyriproxyfen (I)	136 > 78.1 (20)	136 > 96 (15)	320.8 > 222 (10)
30.103	Pyraclostrobin (F)	132 >77.2 (20)	165.5 > 132.9 (10)	132.9 > 104.9 (15)
30.330, 30.631, 32.605	Lambda cyhalothrin (I)	197 > 141 (10)	181 > 152 (25)	197 > 160.9 (5)
30.691	Cypermethrin (I)	163.1 > 90.9 (10)	163.1 > 127.1(5)	165.1 > 90.9 (10)
30.870	Metrafenone (F)	209.1 > 166.1 (10)	395.1 > 365 (15)	227 > 169 (10)
31.246	Spirodiclofen (A)	108.9 > 81 (10)	108.9 > 78.9 (15)	312.2 > 259.1 (10)
31.443	Pyridaben (A)	147.1 > 117 (20)	147.1 > 132.1 (10)	147.1 > 105.1 (10)
31.547	Fluquinconazole (F)	340.1 > 298 (15)	108 > 57 (15)	340.1 > 108 (40)
31.647	Prochloraz (F)	195.9 > 97.1 (30)	180.1 > 138.1 (10)	310 > 70 (15)
32.052	Fenbuconazole (F)	129 > 102 (15)	198.1 > 129 (5)	129 > 78 (20)

32.698	Boscalid (F)	140 > 112 (10)	140 > 76.1 (25)	112 > 76.1 (15)
32.744	Quizalofop-P-ethyl (H)	372.1 > 299 (10)	163 > 136 (10)	163 > 100 (20)
33.001, 34.533	Tau fluvalinate (I)	250 > 55 (40)	180.9 > 152 (40)	250 > 200 (40)
34.313	Imibenkonazol (F)	125.1 > 89 (20)	125.1 > 98.8 (20)	253.2 > 81.9 (5)
34.409, 34.022	Fenvalerate	167 > 125 (5)	209 > 141 (15)	181 > 152 (20)
35.496, 35.062	Deltamethrin (I)	253 > 93.1 (15)	181.1 > 152 (25)	251 > 172 (5)
36.126	Azoxystrobin (F)	344.1 > 330 (15)	344.1 > 172.1 (40)	344.1 > 183.1 (25)
36.697	Famoksadone (F)	197 > 141 (15)	224 > 196 (10)	197 > 115 (30)
37.132	Indoxacarb (I)	203.1 > 134 (15)	203.1 > 106 (25)	203.1 > 78 (30)

I-*insecticides,* F – *fungicides,* H – *herbicides,* A - *acaricides*

Table S2. Single ion monitoring SIM and retention times for PAHs

**Retention time (min)**	**Active substance**	**SIM**
7.27	Acenaphtylene	152.2
8.1	Fluorene	166.2
9.8	Phenanthrene	178.2
9.85	Anthracene	178.2
12.86	Pyrene	202.2
16.03	Benzo[*a*]anthracene	228.2
16.15	Chrysene	228.2
18,96	Benzo[*b*]fluoranthrene	252.2
19.02	Benzo[*k*]fluoranthrene	252.2
19.77	Benzo[*a*]pyrene	252.1
22.58	Indeno[1,2,3-*cd*]pyrene	276.1
22.63	Dibenzo[*a,h*]anthracene	278.1
23.28	Benzo[*ghi*]perylene	276.1
